# A comparison of Monte Carlo-based Bayesian parameter estimation methods for stochastic models of genetic networks

**DOI:** 10.1371/journal.pone.0182015

**Published:** 2017-08-10

**Authors:** Inés P. Mariño, Alexey Zaikin, Joaquín Míguez

**Affiliations:** 1 Departamento de Biología y Geología, Física y Química Inorgánica, Universidad Rey Juan Carlos, Móstoles, Madrid 28933, Spain; 2 Institute for Women’s Health, University College London, London WC1E 6BT, United Kingdom; 3 Department of Mathematics, University College London, London WC1E 6BT, United Kingdom; 4 Department of Applied Mathematics, Lobachevsky State University of Nizhny Novgorod, Nizhniy Novgorod, Russia; 5 Departamento de Teoría de la Señal y Comunicaciones, Universidad Carlos III de Madrid, Leganés, Madrid, Spain; Western University, CANADA

## Abstract

We compare three state-of-the-art Bayesian inference methods for the estimation of the unknown parameters in a stochastic model of a genetic network. In particular, we introduce a stochastic version of the paradigmatic synthetic multicellular clock model proposed by Ullner *et al.*, 2007. By introducing dynamical noise in the model and assuming that the partial observations of the system are contaminated by additive noise, we enable a principled mechanism to represent experimental uncertainties in the synthesis of the multicellular system and pave the way for the design of probabilistic methods for the estimation of any unknowns in the model. Within this setup, we tackle the Bayesian estimation of a subset of the model parameters. Specifically, we compare three Monte Carlo based numerical methods for the approximation of the posterior probability density function of the unknown parameters given a set of partial and noisy observations of the system. The schemes we assess are the particle Metropolis-Hastings (PMH) algorithm, the nonlinear population Monte Carlo (NPMC) method and the approximate Bayesian computation sequential Monte Carlo (ABC-SMC) scheme. We present an extensive numerical simulation study, which shows that while the three techniques can effectively solve the problem there are significant differences both in estimation accuracy and computational efficiency.

## Introduction

The field of systems biology is rich in problems that demand sophisticated computational tools for estimation, detection and prediction. As a consequence, we are witnessing the development of a rigorous engineering discipline to create, control and programme cellular behaviour [1]. The resulting branch of research, known as synthetic biology, has undergone a dramatic growth throughout the past decade and is poised to transform biotechnology and medicine. A core issue in synthetic biology is the analysis of networks of interacting biomolecules [2], which carry out many essential functions in living cells. However, the design principles underlying the functioning of such intracellular networks remain poorly understood. To develop new models and to simplify significantly the associated engineering processes, one needs new inference methods that enable the accurate calibration of potentially complex models by estimating any unknown parameters. It is expected that the ability to design complex synthetic networks will lead both to the engineering of new cellular behaviours and to an improved understanding of naturally occurring networks.

A particular system that has drawn considerable attention is the so-called repressilator [3] which is an oscillating network that periodically induces the synthesis of a green fluorescent protein as a readout of its state in individual cells and can be considered as a synthetic biological clock. Mathematical models, consisting of systems of nonlinear differential equations, that describe the dynamics of the original repressilator and subsequent extensions of it have appeared in the literature [3–9] and sparked interest from researchers in physics, engineering and mathematics.

In this paper we investigate the application of state-of-the-art Bayesian inference methods for the estimation of the unknown parameters in a stochastic model of a genetic network. In particular, we introduce a stochastic version of the chaotic, continuous-time modified repressilator model of [8], which consists of a set of stochastic differential equations (SDEs) driven by Wiener noise processes. These equations depend on a number of unknown parameters, which we model as random variables. We convert the system of SDEs into an (approximate) discrete-time state space model using a standard Euler-Maruyama scheme and then consider the problem of computing the posterior probability distribution of the unknown parameters in the model conditional on a sequence of partial observations that consist of noisy measurements of a small subset of the (dynamic) state variables. This setup resembles the scenario considered in [8] but (i) the system in this paper is stochastic, while in [8] only a deterministic model was studied, and (ii) we pose a data-poor problem, with the collected observations being low dimensional (2-dimensional, for a 14-dimensional state space), noisy and sparse in time, whereas in [8] data were assumed available continuously in time and noise-free. The randomness in the proposed model dynamics can potentially account for experimental uncertainties in the synthesis of the biological system. It also enables the application of probabilistic methods for the calibration of the model and its simulation and forecasting.

Within this probabilistic framework we investigate the Bayesian estimation of the unknown model parameters, i.e., the approximation of their posterior probability distribution conditional on the available observations. In particular, we compare three state-of-the-art Monte Carlo methods for Bayesian inference: the particle Metropolis-Hastings (PMH) method [10], the nonlinear population Monte Carlo (NPMC) algorithm [11] and the approximate Bayesian computation sequential Monte Carlo (ABC-SMC) scheme [8, 12]. PMH is a Markov chain Monte Carlo (MCMC) [13] method that relies on a built-in particle filtering approximation [14] to compute the likelihood of the unknown parameters, which cannot be obtained in closed-form for general nonlinear systems (such as the stochastic repressilator). The algorithm produces a sequence of parameter values that form a Markov chain and the limit probability of this chain is precisely the posterior distribution of the unknown parameters. The NPMC scheme relies on an importance sampling (IS) [15] approximation of the posterior parameter distribution, rather than a Markov chain approach. It employs the same particle filtering approximation of the parameter likelihoods as the PMH method but its key feature is the computation of non-linearly transformed importance weights (unlike conventional IS methods) in order to reduce the variance of the parameter estimates. Preliminary results on the application of the NPMC method to the coupled-repressilator model can be found in the conference communication [16]. Finally, the ABC-SMC algorithm is a likelihood-free procedure that relies on the computation of distances between observed and synthetic data in order to weight candidate values for the unknown parameters.

We have carried out an extensive numerical comparison of the three methods, taking into account both the accuracy of the parameter estimates and their computational cost. We have considered scenarios where

- the signals are generated from a stochastic coupled repressilator schemes and
- noisy observations are collected from a deterministic (conventional) coupled repressilator system.

While the three schemes effectively solve the problem, the NPMC and PMH algorithm attain a clearly smaller estimation error than the ABC-SMC scheme for the same computational cost. When the computational budget is kept low, the PMH and NPMC algorithms attain very similar estimation errors, with some advantage for the PMH method in the stochastic-signal scenario. As we increase the computational effort, the NPMC algorithm outperforms the PMH scheme in our experiments.

In the sequel, we introduce the stochastic modified repressilator model to be investigated and describe the probabilistic inference methods. Then we present and discuss the results of an extensive set of computer simulations.

## Methods

### Intercellular network model

#### Modified stochastic repressilator

**Fig 1:**
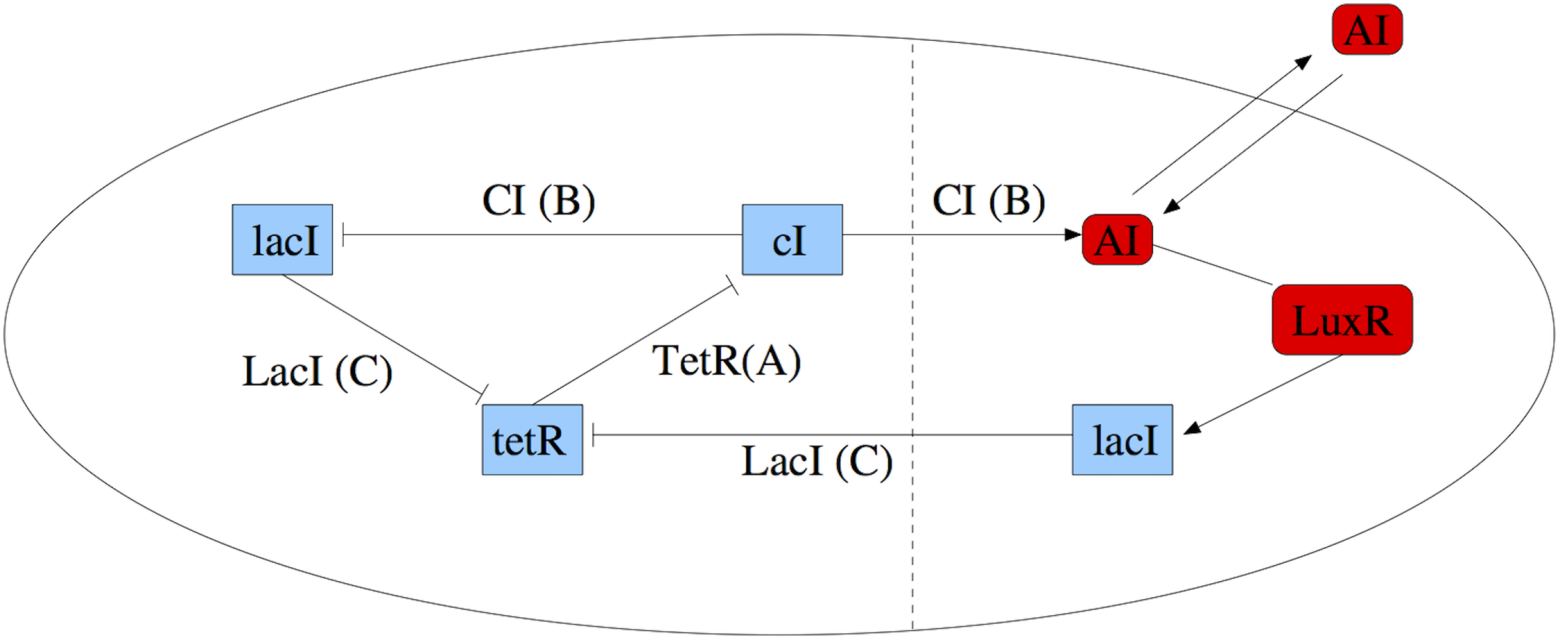
Plot of the modified repressilator. The elements added to the standard repressilator are depicted on the right hand side of the dashed vertical line. Genes and molecules are represented in light blue and red colours, respectively. The oval depicts the cell membrane and the long lines ending in small vertical bars represent the corresponding inhibitory couplings.

The standard repressilator is a “genetic clock” built around three genes, where the protein product of each gene represses the expression of another one in a cyclic manner [3]. It produces nearly harmonic oscillations in protein levels. In the original repressilator design, the gene *lacI* expresses protein LacI, which inhibits transcription of the gene *tetR*. The product of the latter, TetR, inhibits transcription of the gene *cI*. Finally, the protein product CI of the gene *cI* inhibits expression of *lacI* and completes the cycle. We can see this mechanism in the left side of Fig 1, where the genes are represented in light blue colour.

Fig 1 represents a modification of the repressilator, introduced in [5], that includes an additional feedback loop involving a small autoinducer (AI) molecule produced by CI that can diffuse through the cell membrane, and the protein LuxR, which responds to the AI by activating the transcription of a second copy of the repressilator gene *lacI*. Placing the gene *cI* under inhibitory control of the repressilator protein TetR leads to a repressive and phase-repulsive coupling that, in turn, generates rich dynamical patterns, including chaotic oscillations [5]. Phase repulsive coupling is common in many biological systems, e.g., in neural activity, in the brain of songbirds or in the respiratory system.

In this paper we study a model consisting of two modified repressilators with identical parameters, driven by Wiener-type noise and coupled by the fast diffusion of the AI across the cell membranes. The resulting mRNA dynamics in continuous time t∈R is described by a stochastic Hill-type equation with coefficient m, namely

$$\begin{array}{ccc} da_{i} & = & -\left(a_{i}-\frac{\alpha }{1+C_{i}^{m}}\right)dt+\sigma_{a}a_{i}dW_{i}^{a}, \end{array}$$ (1)

$$\begin{array}{ccc} db_{i} & = & -\left(b_{i}-\frac{\alpha }{1+A_{i}^{m}}\right)dt+\sigma_{b}b_{i}dW_{i}^{b}, \end{array}$$ (2)

$$\begin{array}{ccc} dc_{i} & = & -\left(c_{i}-\frac{\alpha }{1+B_{i}^{m}}-\frac{\kappa S_{i}}{1+S_{i}}\right)dt+\sigma_{c}c_{i}dW_{i}^{c}, \end{array}$$ (3)

where the subscript *i* = 1, 2 specifies the cell; *a*_*i*_, *b*_*i*_, and *c*_*i*_ are time-varying state variables (stochastic processes) representing the concentrations of mRNA molecules transcribed from the genes of *tetR*, *cI*, and *lacI*, respectively; the constant parameter *α* is the dimensionless transcription rate in the absence of a repressor; the constant parameter *κ* is the maximum transcription rate of the LuxR promoter; *S*_*i*_ is a state variable representing the concentration of the AI molecule inside cell *i*, and $W_{i}^{a}$, $W_{i}^{b}$, $W_{i}^{c}$, *i* = 1, 2, are independent standard Wiener processes scaled by the constant non-negative factors *σ*_*a*_, *σ*_*b*_ and *σ*_*c*_, respectively. The additional time-varying states *A*_*i*_, *B*_*i*_, and *C*_*i*_, *i* = 1, 2, are stochastic processes representing the concentration of the proteins TetR, CI, and LacI, respectively, whose dynamics obey the SDEs

$$\begin{array}{ccc} dA_{i} & = & \beta_{a}\left(a_{i}-A_{i}\right)dt+\sigma_{A}A_{i}dW_{i}^{A}, \end{array}$$ (4)

$$\begin{array}{ccc} dB_{i} & = & \beta_{b}\left(b_{i}-B_{i}\right)dt+\sigma_{B}B_{i}dW_{i}^{B}, \end{array}$$ (5)

$$\begin{array}{ccc} dC_{i} & = & \beta_{c}\left(c_{i}-C_{i}\right)dt+\sigma_{C}C_{i}dW_{i}^{C}. \end{array}$$ (6)

The equations above show that the dynamics of the proteins is linked to the amount of the responsible mRNA, and the constant parameters *β*_*a*_, *β*_*b*_ and *β*_*c*_ describe the ratio between mRNA and the protein lifetimes (i.e, the inverse degradation rates). Similar to Eqs (1)–(3), the dynamics is driven by independent standard Wiener processes $W_{i}^{A}$, $W_{i}^{B}$ and $W_{i}^{C}$, *i* = 1, 2, with constant scale factors *σ*_*A*_, *σ*_*B*_, *σ*_*C*_ ≥ 0. The model is made dimensionless by measuring time in units of the mRNA lifetime (assumed equal for all genes) and the mRNA and protein levels in units of their Michaelis constant. The mRNA concentrations are additionally rescaled by the ratio of their protein degradation and translation rates [4, 5].

The term $\frac{\kappa S_{i}}{1+S_{i}}$ on the right-hand side of Eq (3) represents activated production of *lacI* by the AI molecule, whose concentration inside cell *i* is denoted by *S*_*i*_. The dynamics of CI and LuxI can be considered identical, given that their production is controlled by the same protein (TetR). Hence, the synthesis of the AI is controlled by the concentration *B*_*i*_ of the protein CI. Taking also into account the intracellular degradation of the AI and its diffusion, the dynamics of *S*_*i*_ is modelled as

$$\begin{array}{c} dS_{i}=-\left(k_{s0}S_{i}-k_{s1}B_{i}+\eta \left(S_{i}-S_{e}\right)\right)dt+\sigma_{S}S_{i}dW_{i}^{S}, \end{array}$$ (7)

where *k*_*s*0_, *k*_*s*1_ and *η* are constant parameters, the latter being a diffusion coefficient that depends on the permeability of the membrane to the AI. The variable *S*_*e*_ is the extracellular concentration of the AI molecule. It is common to apply a quasi-steady-state approximation to the dynamics of *S*_*e*_ [4, 5], which leads to $S_{e}=Q\bar{S}\equiv Q\frac{1}{N}\sum_{i=1}^{N}S_{i},$ where $Q=\frac{\delta N}{V_{ext}\left(k_{se}+\frac{\delta N}{V_{ext}}\right)}$, *N* = 2 is the number of cells, *V*_*ext*_ is the total extracellular volume, *k*_*se*_ is the extracellular AI degradation rate, and *δ* is the product of the membrane permeability and the surface area.

This model can produce a range of dynamic regimes. We achieve an underlying chaotic behaviour for this model when the constant parameters are set as [5]

$$\begin{array}{c} \left(Q,m,\alpha ,\beta_{a},\beta_{b},\beta_{c},\eta ,\kappa ,k_{s0},k_{s1}\right)=\left(0.85,2.6,216,0.85,0.1,0.1,2,25,1,0.01\right). \end{array}$$ (8)

We will refer to these values as standard. Note that we focus on a system with underlying chaotic dynamics because this makes the parameter estimation problem more challenging. Due to the system sensitivity to small variations of the parameters, two choices of parameter sets, one closer than the other to the standard values, may appear equally “incompatible” with a sequence of observations produced by a system endowed with the standard parameters. Methods that work well in the chaotic regime can be expected to work well in other scenarios.

#### Numerical integration and state space model

In order to integrate the 14-dimensional SDE described by Eqs (1)–(7) numerically, we apply the Euler-Maruyama discretisation with integration step h<<1, that can be explicitly written as

$$\begin{array}{ccc} a_{i,m+1} & = & a_{i,m}-h\left(a_{i,m}-\frac{\alpha }{1+C_{i,m}^{m}}\right)+\sigma_{a}a_{i,m}w_{i,m}^{\left(1\right)}, \end{array}$$ (9)

$$\begin{array}{ccc} b_{i,m+1} & = & b_{i,m}-h\left(b_{i}\left(n\right)-\frac{\alpha }{1+A_{i,m}^{m}}\right)+\sigma_{b}b_{i,m}w_{i,m}^{\left(2\right)}, \end{array}$$ (10)

$$\begin{array}{ccc} c_{i,m+1} & = & c_{i,m}-h\left(c_{i,m}-\frac{\alpha }{1+B_{i,m}^{m}}-\frac{\kappa S_{i,m}}{1+S_{i,m}}\right)+\sigma_{c}c_{i,m}w_{i,m}^{\left(3\right)}, \end{array}$$ (11)

$$\begin{array}{ccc} A_{i,m+1} & = & A_{i,m}+h\beta_{a}\left(a_{i,m}-A_{i,m}\right)+\sigma_{A}A_{i,m}w_{i,m}^{\left(4\right)}, \end{array}$$ (12)

$$\begin{array}{ccc} B_{i,m+1} & = & B_{i,m}+h\beta_{b}\left(b_{i,m}-B_{i,m}\right)+\sigma_{B}B_{i,m}w_{i,m}^{\left(5\right)}, \end{array}$$ (13)

$$\begin{array}{ccc} C_{i,m+1} & = & C_{i,m}+h\beta_{c}\left(c_{i,m}-C_{i,m}\right)+\sigma_{C}C_{i,m}w_{i,m}^{\left(6\right)}, \end{array}$$ (14)

$$\begin{array}{ccc} S_{i,m+1} & = & S_{i,m}-h\left(k_{s0}S_{i,m}-k_{s1}B_{i}\left(n\right)+\eta \left(S_{i,m}-S_{e,m}\right)\right)+\sigma_{S}S_{i,m}w_{i,m}^{\left(7\right)}, \end{array}$$ (15)

where *i* = 1, 2 and $\left\{w_{i,m}^{\left(1\right)},\ldots ,w_{i,m}^{\left(7\right)}\right\}$ are independent Gaussian random variables (r.v.’s) with zero mean and variances $\sigma_{a}^{2},\sigma_{b}^{2},\sigma_{c}^{2},\sigma_{A}^{2},\sigma_{B}^{2},\sigma_{C}^{2}$ and $\sigma_{S}^{2}$, respectively.

The system described by Eqs (9)–(15) can be compactly written as the multidimensional difference equation

$$\begin{array}{c} {\bar{x}}_{m}=F_{\theta }\left({\bar{x}}_{m-1},w_{m}\right), \end{array}$$ (16)

where $F_{\theta }:R^{14}\to R^{14}$ is a function that accounts for both the deterministic and the stochastic part of the model and depends on a vector of unknown parameters *θ* (modelled as random), ${\bar{x}}_{m}={\left[{\bar{x}}_{1,m}^{⊤},{\bar{x}}_{2,m}^{⊤}\right]}^{⊤}$ is the 14 × 1 state of the system at discrete time m∈Z, ${\bar{x}}_{i,m}$ are the 7 × 1 state vectors associated to the two cells, *i* = 1, 2, and $w_{m}={\left[w_{1,m}^{⊤},w_{2,m}^{⊤}\right]}^{⊤}$ is an independent and identically distributed (i.i.d.) sequence of 14 × 1 zero-mean Gaussian vectors. Each 7 × 1 subvector **w**_*i*,*m*_, *i* = 1, 2, has the same distribution and can be written as $w_{i,m}={\left[w_{i,m}^{\left(1\right)},\ldots ,w_{i,m}^{\left(7\right)}\right]}^{⊤}$. In particular, note that, for each cell,

$$\begin{array}{c} {\bar{x}}_{i,m}={\left[a_{i,m},b_{i,m},c_{i,m},A_{i,m},B_{i,m},C_{i,m},S_{i,m}\right]}^{⊤}, \end{array}$$ (17)

with the continuous-time state variables evaluated at time t=mh, e.g., $a_{i,m}=a_{i}\left(t=mh\right)$. The same as in [8], all constant parameters are assumed known except $\theta ={\left[Q,m,\alpha ,\beta_{a}\right]}^{⊤}$, which are unknown and modelled as r.v.’s. We assume uniform and independent a priori probability distributions for each one of these parameters, namely Q∼U(0,1), m∼U(1,5), α∼U(50,300) and $\beta_{a}∼U\left(0,1\right)$. The parameter vector *θ*, therefore, takes values on the set S=(0,1)×(1,5)×(50,300)×(0,1). We denote the conditional (on *θ*) Markov kernel that determines the state transition from time *m* − 1 to time *m* as ${\bar{K}}_{\theta }\left(dx_{m}|x_{m-1}\right)$. In particular, for a Borel set $A\subset R^{14}$, ${\bar{K}}_{\theta }\left(A|x_{m-1}\right)$ is the probability of moving from the point ${\bar{x}}_{m-1}$ in the state space to some ${\bar{x}}_{m}\in A$.

Partial and noisy observations of the system are collected every *m*_0_ discrete time steps, i.e., every $t_{o}=m_{0}h$ continuous time units. Only the variables *a*_*i*_, *i* = 1, 2, are observable, hence the observations have the form

$$\begin{array}{c} y_{n}=\left[\begin{array}{c} a_{1,nm_{o}}\\ a_{2,nm_{o}} \end{array}\right]+\sigma_{y}\epsilon_{n},\;n=1,2,... \end{array}$$ (18)

where ***ϵ***_*n*_ is a sequence of independent 2 × 1 Gaussian random vectors (with zero mean and identity covariance matrix) and *σ*_*y*_ > 0 is a known constant parameter that determines the noise power.

In order to put the states and the observations on the same time scale, we define the sequence of states {**x**_*n*_}_*n*≥0_ as $x_{n}≜{\bar{x}}_{nm_{0}}$ and introduce the composite Markov kernel

$$\begin{array}{c} K_{\theta }\left(dx_{n}|x_{n-1}\right)={\bar{K}}_{\theta }\left(dx_{n}|{\bar{x}}_{nm_{0}-1}\right){\bar{K}}_{\theta }\left(d{\bar{x}}_{nm_{0}-1}|{\bar{x}}_{nm_{0}-2}\right)\cdots {\bar{K}}_{\theta }\left(d{\bar{x}}_{\left(n-1\right)m_{0}+1}|x_{n-1}\right). \end{array}$$ (19)

For a Borel set $A\subset R^{14}$, $K_{\theta }\left(A|x_{n-1}\right)$ is the probability of the event **x**_*n*_ ∈ *A* (where $x_{n}={\bar{x}}_{nm_{0}}$) conditional on $x_{n-1}={\bar{x}}_{\left(n-1\right)m_{0}}$ (and on the parameter vector *θ*).

The pair of sequences **x**_*n*_ and **y**_*n*_ yield a discrete-time, Markov state space model [17] conditional on the choice of parameters *θ*. The model is specified by the prior probability distribution of the state **x**_0_, which we denote as $K_{0}\left(dx_{0}\right)$, the dynamics of the state sequence **x**_*n*_, which is given by the Markov kernel $K_{\theta }\left(dx_{n}|x_{n-1}\right)$, and the conditional pdf of the observations **y**_*n*_ given the states **x**_*n*_, which we denote as *l*_*n*_(**y**_*n*_|**x**_*n*_). We note that, in this model, the latter density is independent of the parameters *θ*. Also, since **y**_*n*_ − [*a*_1,*nm*_0__, *a*_2,*nm*_0__]^⊤^ = ***ϵ***_*n*_, the form of the function *l*_*n*_(**y**_*n*_|**x**_*n*_) is given by the pdf of the noise term ***ϵ***_*n*_. We often refer to *l*_*n*_(**y**_*n*_|**x**_*n*_) as the likelihood of the state **x**_*n*_.

### Algorithms

In a Bayesian probabilistic setup, the unknown parameters are modelled as a random vector and the aim is to approximate its posterior probability distribution, conditional on the available observations **y** = {**y**_1_, **y**_1_, …, **y**_*R*_} for some fixed *R* > 0. We denote the posterior pdf of the unknown parameters as *p*(*θ*|**y**) and note that it can be readily factored, using Bayes’ theorem, as *p*(*θ*|**y**) ∝ *ℓ*(**y**|*θ*)*p*_0_(*θ*), where *ℓ*(**y**|*θ*) is proportional to the conditional pdf of the observations **y** given the parameters *θ* (i.e., the likelihood of *θ*) and *p*_0_(*θ*) is the prior density of *θ*, which has been chosen to be uniform on the set S, as described in the previous section.

For the case of general state space models, an additional difficulty encountered when trying to estimate the unknown model parameters (denoted *θ* in our setup) is that the likelihood *ℓ*(**y**|*θ*) is intractable. In the last few years, though, it has become a common approach to approximate this likelihood via particle filtering (PF) (see, e.g., [10, 11, 18–20]). To be specific, we let *ℓ*^*N*^(**y**|*θ*) stand for the approximation of *ℓ*(**y**|*θ*) computed using a standard bootstrap filter (BF) [21, 22] with *N* particles (see S1 Appendix for full details). One key feature of this approach is that *ℓ*^*N*^(**y**|*θ*) can be proved to be an unbiased estimator of *ℓ*(**y**|*θ*) [23].

#### Particle Metropolis-Hastings (PMH)

The PMH is a member of the class of particle MCMC methods [10] that have become very popular in recent years. It can be seen as a conventional Metropolis-Hastings algorithm [15] where the likelihood of each candidate value of *θ* is approximated via particle filtering and, hence, its acceptance probability is also approximate. Given a Markov kernel $M(\theta^{\prime }|\theta )$, the PMH algorithm generates a chain on the space of the parameter *θ* as follows:

1. Initialisation: Draw *θ*_0_ ∼ *p*_0_(*θ*) from the prior distribution of the parameters.
2. At the *m*-th iteration, and given the previous sample *θ*_*m*−1_:
  - Draw a tentative new element ${\tilde{\theta }}_{m}$ from the kernel $M(\theta |\theta_{m-1})$.
  - Compute the (approximate) likelihood $\ell^{N}\left(y|{\tilde{\theta }}_{m}\right)$ and prior density $p_{0}\left({\tilde{\theta }}_{m}\right)$. The acceptance probability for ${\tilde{\theta }}_{m}$ is
    $$\begin{array}{c} \alpha_{m}=\min\left(1,\frac{\ell^{N}\left(y|{\tilde{\theta }}_{m}\right)p_{0}\left(\theta_{m-1}\right)M\left({\tilde{\theta }}_{m}|\theta_{m-1}\right)}{\ell^{N}\left(y|\theta_{m-1}\right)p_{0}\left({\tilde{\theta }}_{m}\right)M\left(\theta_{m-1}|{\tilde{\theta }}_{m}\right)}\right) \end{array}$$ (20)
  - Draw $u_{m}∼U\left(0,1\right)$. If *u*_*m*_ < *α*_*m*_ then $\theta_{m}={\tilde{\theta }}_{m}$, else *θ*_*m*_ = *θ*_*m*−1_.

We use this procedure to generate a chain of length *L*, *θ*_0_, *θ*_1_, …, *θ*_*L*−1_. It can be proved that, as *n* → ∞, the probability distribution of *θ*_*n*_ converges to the posterior pdf *p*(*θ*|**y**) [10] despite the approximation *ℓ*^*N*^(**y**|*θ*) ≃ *ℓ*(**y**|*θ*) in the algorithm. Since the chain requires a number of iterations to converge to its limit distribution (this is often referred to in the literature as the “burn in” period), in practice we need to discard some samples at the beginning of the chain. Herein we assume that we use the second half of the chain to estimate *θ*. In other words, if we aim at approximating the expected value of *θ* given the observations, often denoted *E*[*θ*|**y**], we approximate it as the sample mean of *θ*_*L*/2_, …, *θ*_*L*−1_, i.e.

$$\begin{array}{c} {\hat{\theta }}_{L}=\frac{2}{L}\sum_{i=L/2}^{L-1}\theta_{i} \end{array}$$ (21)

where we assume, for convenience, that *L* is even. Note that we can also approximate other posterior statistics of *θ* (given **y**). For eample, the posterior covariance of *θ* can be estimated as

$$\begin{array}{c} {\hat{\Sigma }}_{L}=\frac{2}{L}\sum_{i=L/2}^{L-1}\left(\theta_{i}-{\hat{\theta }}_{L}\right){\left(\theta_{i}-{\hat{\theta }}_{L}\right)}^{T}. \end{array}$$ (22)

#### Nonlinear population Monte Carlo (NPMC)

The NPMC algorithm of [11] is an iterative importance sampling (IS) scheme that seeks to approximate a target probability distribution, in our case given by the pdf *p*(*θ*|**y**), using weighted Monte Carlo samples. This algorithm generates a sequence of proposal pdf’s *q*_*k*_(*θ*), *k* = 1, …, *K*, from which samples can be drawn and importance weights (IWs) can be computed. This sequence of proposals is expected to yield increasingly better approximations of the target as the algorithm converges. The key feature of the NPMC method, which departs from the classical PMC technique of [24], is to compute a set of *transformed* importance weights (TIWs) by applying a nonlinear function to the standard IWs. The aim of this transformation is to mitigate the well known problem of the degeneracy of the IWs (common to many IS methods, see [11, 25]) by controlling the weight variability. Some basic results regarding the convergence in probability of estimators based on TIWs are given in [11]. A specific analysis for the case of state space models, where the likelihood function *ℓ*(**y**|*θ*) can only be estimated via particle filtering, can be found in [26].

The NPMC algorithm with *K* iterations, *M* Monte Carlo samples per iteration, plain Gaussian proposals {*q*_*k*_}_*k*≥1_, and approximate likelihoods is outlined below. Recall that *θ* is the vector of unknown model parameters.

***Initialisation***. Draw *M* i.i.d. samples $\theta_{0}^{1},\theta_{0}^{2},\ldots ,\theta_{0}^{M}$ from the prior pdf *p*_0_(*θ*). Then,

1. compute non-normalised IWs ${\tilde{w}}_{0}^{i}\propto \ell^{N}\left(y|\theta_{0}^{i}\right)$, *i* = 1, …, *M*,
2. compute TIWs as ${\hat{w}}_{0}^{i}=T_{M}\left(i,{\left\{{\tilde{w}}_{0}^{j}\right\}}_{j=1}^{M}\right)$, where $T_{M}:\left\{1,\ldots ,M\right\}\times {\left\{{\tilde{w}}_{0}^{j}\right\}}_{j=1}^{M}\to \left[0,+\infty \right)$ is a nonlinear map (to be specifically described below),
3. normalise the TIWs, $w_{0}^{i}=\frac{{\hat{w}}_{0}^{i}}{\sum_{j=1}^{M}{\hat{w}}_{0}^{j}}$, *i* = 1, …, *M*.

***Iterative step***. For *k* = 1, …, *K*, take the following steps:

1. Let $q_{k}\left(\theta \right)=N\left(\theta |\mu_{k},\Sigma_{k}\right)$ be a multivariate Gaussian pdf with mean vector and covariance matrix obtained, respectively, as
  $$\begin{array}{c} \mu_{k}=\sum_{i=1}^{M}w_{k-1}^{i}\theta_{k-1}^{i}\;\text{and}\;\Sigma_{k}=\sum_{i=1}^{M}w_{k-1}^{i}\left(\theta_{k-1}^{i}-\mu_{k}\right){\left(\theta_{k-1}^{i}-\mu_{k}\right)}^{⊤}. \end{array}$$ (23)
  Note that the random variates $\theta_{k-1}^{i}$, *i* = 1, …, *M*, are 4 × 1 vectors.
2. Draw $\theta_{k}^{i}$, *i* = 1, …, *M*, i.i.d. samples from *q*_*k*_(*θ*).
3. Compute IWs, ${\tilde{w}}_{k}^{i}=\frac{\ell^{N}\left(y|\theta_{k}^{i}\right)p_{0}\left(\theta_{k}^{i}\right)}{q_{k}\left(\theta_{k}^{i}\right)}$, *i* = 1, …, *M*.
4. Compute TIWs, ${\hat{w}}_{k}^{i}=T_{M}\left(i,{\left\{{\tilde{w}}_{k}^{j}\right\}}_{j=1}^{M}\right)$, *i* = 1, …, *M*, using the same nonlinear map as for *k* = 0.
5. Normalise the TIWs, $w_{k}^{i}=\frac{{\hat{w}}_{k}^{i}}{\sum_{j=1}^{M}{\hat{w}}_{k}^{j}}$, *i* = 1, …, *M*.

Following [11], the nonlinear map $T_{M}$ of choice is a “clipping” transformation. In particular, let *i*_1_, *i*_2_, …, *i*_*M*_ be a permutation of the indices 1, 2, …, *M* such that the IWs become ordered, namely ${\tilde{w}}_{k}^{i_{1}}\geq {\tilde{w}}_{k}^{i_{2}}\geq \cdots \geq {\tilde{w}}_{k}^{i_{M}}$. The clipping transformation $T_{M}$, with parameter $1\leq M_{c}\leq \sqrt{M}$, flattens the *M*_*c*_ largest IWs and makes them equal to the *M*_*c*_-th non-normalised IW, ${\tilde{w}}_{k}^{i_{M_{c}}}$. Specifically, for each *i* = 1, …, *M*, we obtain

$$\begin{array}{c} {\hat{w}}_{k}^{j}=T_{M}\left(j,{\left\{{\tilde{w}}_{k}^{l}\right\}}_{l=1}^{M}\right)=\{\begin{array}{cc} {\tilde{w}}_{k}^{i_{M_{c}}}, & \text{if}\;{\tilde{w}}_{k}^{j}\geq {\tilde{w}}_{k}^{M_{c}},\\ {\tilde{w}}_{k}^{j}, & \text{if}\;{\tilde{w}}_{k}^{j}<{\tilde{w}}_{k}^{M_{c}}, \end{array}. \end{array}$$ (24)

Other choices of $T_{M}$ are possible (e.g., tempering schemes [11]).

At any iteration *k* of the algorithm we may use the samples ${\left\{\theta_{k}^{i}\right\}}_{i=1}^{M}$ and the normalised IWs or TIWs for estimating *θ* or approximating any of its statistics. Hereafter, we assume that the normalised TIWs ${\left\{w_{k}^{i}\right\}}_{i=1}^{M}$ are employed. Hence, the expected value of *θ* given the observations **y** is estimated (at the *k*-th iteration) as

$$\begin{array}{c} {\hat{\theta }}_{k}^{M}=\sum_{i=1}^{M}w_{k}^{i}\theta_{k}^{i}≃E\left[\theta |y\right] \end{array}$$ (25)

where the superscript *M* indicates the number of samples used in the estimates. Similarly, the covariance matrix of *θ* conditional on the data **y** can be approximated as

$$\begin{array}{c} {\hat{\Sigma }}_{k}^{M}=\sum_{i=1}^{M}w_{k}^{i}\left(\theta_{k}^{i}-{\hat{\theta }}_{k}^{M}\right){\left(\theta_{k}^{i}-{\hat{\theta }}_{k}^{M}\right)}^{T}. \end{array}$$ (26)

#### Approximate Bayesian computation sequential Monte Carlo (ABC-SMC)

ABC methods have been conceived with the aim of approximating posterior probability distributions, conditional on the available observations, without having to calculate likelihood functions [12, 27]. The computation of the likelihood is replaced by a comparison between simulated data and actual observations. The ABC principle involves three general steps:

- Draw random candidate values for the parameter vector, say *θ*^1^, *θ*^2^, …, from the prior distribution of the parameters *p*_0_(*θ*),
- Use these candidates to simulate synthetic realisations of the observable variables, **y**(*θ*^1^), **y**(*θ*^2^), …, by means of the dynamic model equations and taking a fixed initial condition.
- Compare the actual data (observations) and the synthetic data, **y**(*θ*^1^), **y**(*θ*^2^), … using some suitable distance *d*, i.e., evaluate *d*(**y**, **y**(*θ*^1^)), *d*(**y**, **y**(*θ*^2^)), …

Samples that yield a small enough distance, typically below a prescribed tolerance, *ϵ*, are accepted, and those yielding large distances are discarded. Random candidates are generated and tried until a prescribed number of them are accepted.

The sequential Monte Carlo (SMC) version of ABC is a more sophisticated sampling algorithm that generates a sequence of “populations”, i.e., sets of randomly generated parameter values. Each population is associated to prescribed tolerance on the deviation between the actual and synthetic observations. Hence, for a ABC-SMC algorithm generating *T* populations with *J* members each we need to specify tolerances *ϵ*_1_, *ϵ*_2_, …, *ϵ*_*T*_ and the candidate parameter vectors accepted in the *t*-th population, denoted ${\left\{\theta_{t}^{j}\right\}}_{j=1}^{J}$, all share the feature $d\left(y,y\left(\theta_{t}^{j}\right)\right)<\epsilon_{t}$. If the difference *ϵ*_*t*−1_ − *ϵ*_*t*_ > 0 is small, the intuition is that it should not be hard to generate ${\left\{\theta_{t}^{j}\right\}}_{j=1}^{J}$ from ${\left\{\theta_{t-1}^{j}\right\}}_{j=1}^{J}$. Afther the *T*-th population is generated, we expect to have a population ${\left\{\theta_{T}^{j}\right\}}_{j=1}^{J}$ such that $d\left(y,y\left(\theta_{T}^{j}\right)\right)<\epsilon_{T}$ for all *j*.

The ABC-SMC algorithm generating a sequence of *T* populations, *J* samples per population, with tolerances *ϵ*_1_, *ϵ*_2_, …, *ϵ*_*T*_ and Markov kernel *K*_*t*_(.|.), *t* = 1, …, *T*, is outlined below.

1. Initialisation: set the population indicator *t* = 1 and the sample indicator *j* = 1. Select the initial condition **x**_0_.
2. If *t* = 1, draw *θ*^⋆^ from the prior *p*_0_(*θ*). Else, for *t* > 1 draw *θ*^⋆^ from the mixture density
  $$\begin{array}{c} g_{t}\left(\theta \right)=\sum_{j=1}^{J}w_{t-1}^{j}K_{t}\left(\theta |\theta_{t-1}^{j}\right) \end{array}$$ (27)
  where *K*_*t*_(.|*θ*′) is a symmetric kernel centred around *θ*′ and $w_{t-1}^{1},\ldots ,w_{t-1}^{J}$ are importance weights such that $\sum_{j=1}^{J}w_{t-1}^{j}=1$.
3. If *p*_0_(*θ*^⋆^) = 0, then the parameter *θ*^⋆^ is off the support set *S*. Return to step 2.
4. Simulate a dataset **y**(*θ*^⋆^) from the state Eqs (9)-(15) with initial condition **x**_0_.
5. If *d*(**y**, **y**(*θ*^⋆^)) ≥ *ϵ*_*t*_ reject *θ*^⋆^ and return to step 2.
6. Otherwise, if *d*(**y**, **y**(*θ*^⋆^)) < *ϵ*_*t*_, set $\theta_{t}^{j}=\theta^{⋆}$ and compute the weight
  $$\begin{array}{c} {\tilde{w}}_{t}^{j}=\{\begin{array}{c} 1,\text{if}\;t=1\\ \frac{p_{0}\left(\theta_{t}^{j}\right)}{\sum_{l=1}^{J}w_{t-1}^{l}K_{t}\left(\theta_{t}^{j}|\theta_{t-1}^{l}\right)},\text{if}\;t>0 \end{array} \end{array}$$ (28)
7. If *j* < *J*, set *j* = *j* + 1 and go to step 2.
8. Normalize the weights: $w_{t}^{j}=\frac{{\tilde{w}}_{t}^{j}}{\sum_{l=1}^{J}{\tilde{w}}_{t}^{l}}$, *j* = 1, …, *J*.
9. If *t* < *T*, then set *t* = *t* + 1, set *j* = 1 and return to step 2. Otherwise stop.

The posterior estimate of *θ* is computed as

$$\begin{array}{c} {\hat{\theta }}_{T,J}^{ABC}=\sum_{j=1}^{J}w_{T}^{j}\theta_{T}^{j}. \end{array}$$ (29)

We remark that

- The algorithm performance can be optimised by tuning the number of populations, *T*, and the tolerances *ϵ*_1_ > *ϵ*_2_ > … > *ϵ*_*T*_. However, the latter are limited by the power of the observation noise, $\sigma_{y}^{2}$. For example, if $d\left(y,y\left(\theta^{⋆}\right)\right)=\frac{1}{n}\sum_{k=0}^{n}{\left|\right|y_{k}-y_{k}\left(\theta^{⋆}\right)\left|\right|}^{2}$ and we assume that the real data has been produced using exactly *θ*^⋆^, then $d\left(y,y\left(\theta^{⋆}\right)\right)\to 2\sigma_{y}^{2}$ as *n* → ∞.
- The running time of the ABC-SMC algorithm is random, because of the accept/reject steps 3 and 5. To avoid that the algorithm get stalled at step 2 (sampling), in practice it is common to set a maximum number of draws that can be tried before proceeding to the next population. This implies that some populations may have less than *J* samples.

## Results and discussion

We have carried out computer simulations to assess both the proposed model, i.e., the stochastic modified repressilator model described by Eqs (9)–(15), and the performance of the Monte Carlo based Bayesian inference methods. For all the simulations presented here, the true model parameters are set to their standard values (see Eq (8)) in order to generate synthetic (i.e., simulated) trajectories for the dynamic variables, ${\bar{x}}_{i,m}$, *i* = 1, 2 and *m* = 0, 1, …, and sequences of synthetic observations **y**_*n*_, *n* = 1, 2, …, according to Eq (18). This choice of parameters yields an underlying chaotic dynamics of the state variables, as will be shown below. The integration step of the Euler-Mayurama scheme is $h=10^{-3}$ time units. When needed, observations are generated every *m*_*o*_ = 20 discrete-time steps of model Eqs (9)–(15) (equivalently, every $m_{o}h=0.02$ continuous time units). The observational noise ***ϵ***_*n*_ in Eq (18) is assumed to be zero-mean Gaussian with identity covariance matrix $I_{2}=\left[\begin{array}{cc} 1 & 0\\ 0 & 1 \end{array}\right]$, and the standard deviation parameter is *σ*_*y*_ = 1.

All the computer experiments to be presented here have been carried out using Matlab R2016a (9.0.0.341360, 64 bits) running on an Apple iMac equipped with a 4 GHz quad-core IntelCore i7 (turbo boost up to 4.2 GHz) and 32 GB of RAM.

**Fig 2:**
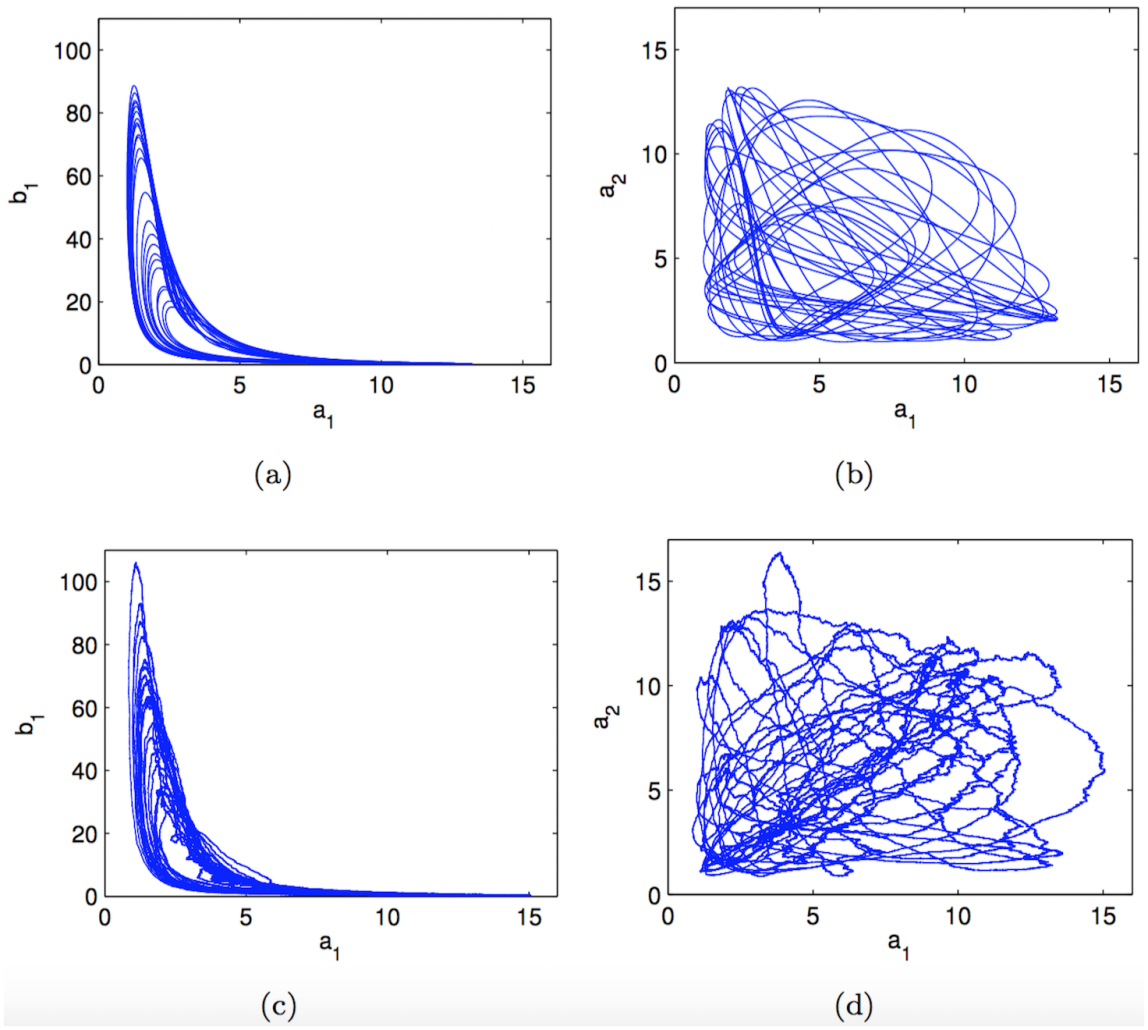
Comparison of 2-dimensional phase space diagrams for the deterministic and the stochastic repressilator models. (a) *b*_1_ versus *a*_1_ and (b) *a*_2_ versus *a*_1_ for the deterministic model; (c) *b*_1_ versus *a*_1_ and (d) *a*_2_ versus *a*_1_ for the stochastic model with common variance 0.02^2^ for the dynamical noise in every state equation.

When the parameters *σ*_*a*_, *σ*_*b*_, *σ*_*c*_, *σ*_*A*_, *σ*_*B*_, *σ*_*C*_ and *σ*_*S*_, which control the variance of the process noise variables $w_{i,m}^{\left(j\right)}$, *j* = 1, …, 7, *i* = 1, 2, are set to zero, we recover the deterministic modified repressilator of [8], which displays chaotic behaviour. For this case, we have run a long simulation of the noiseless system (10,000 continuous time units) and used the results to obtain phase diagrams. In particular, Fig 2(a) shows the phase diagram for the variable *b*_1_ versus *a*_1_, while Fig 2(b) depicts the phase diagram of *a*_2_ versus *a*_1_. What we observe are two views of the multidimensional chaotic attractor generated by this system. When we add dynamical noise in the state equations Eqs (9)–(15), by setting $\sigma_{a}^{2}=\sigma_{b}^{2}=\sigma_{c}^{2}=\sigma_{A}^{2}=\sigma_{B}^{2}=\sigma_{C}^{2}=\sigma_{S}^{2}=0.02^{2}$, we obtain an stochastic dynamical system. However, if we repeat the experiment to generate long trajectories (with the same initial conditions and the same duration) we obtain two similar phase diagrams, as shown in Fig 2(c) and 2(d). Indeed, these figures simply depict perturbed versions of the original deterministic attractor. This illustrates the fact that the underlying chaotic dynamics is preserved in the stochastic model, which can account for slight perturbations or uncertainties in the physical system as well.

In the sequel, we compare the performance of the NPMC, PMH and the ABC-SMC parameter estimation methods described in the *Algorithms* section above. For the first set of computer experiments, we simulate trajectories of the coupled stochastic repressilator model in Eqs (9)–(15) with dynamical noise variance $\sigma_{a}^{2}=\sigma_{b}^{2}=\sigma_{c}^{2}=\sigma_{A}^{2}=\sigma_{B}^{2}=\sigma_{C}^{2}=\sigma_{S}^{2}=0.05^{2}$ and random initial conditions. To be specific, the initial condition of each simulated trajectory is drawn from a multivariate Gaussian distribution with mean

$$\begin{array}{ccc} \left(a_{1,0},b_{1,0},c_{1,0},A_{1,0},B_{1,0},C_{1,0},S_{1,0},a_{2,0},b_{2,0},c_{2,0},A_{2,0},B_{2,0},C_{2,0},S_{2,0}\right) & = & \\ \left(4.5,6,3,4.2,19,4.3,0.1,7.3,1.5,3.4,7,6.5,3.6,0.08\right) & & \end{array}$$ (30)

and covariance matrix $\sigma_{0}^{2}I$, where $\sigma_{0}^{2}=0.05^{2}$. Once the system state trajectory is generated, we produce synthetic observations over an interval of 80 continuous time units (which amounts to $80/h=80\times 10^{3}$ time steps in the Euler-Maruyama scheme). Since observations are assumed to be collected every *m*_*o*_ = 20 discrete steps, this yields a sequence of 4 × 10^3^ 2-dimensional observation vectors contaminated with zero mean Gaussian noise with unit variance (namely, $\sigma_{y}^{2}=1$). In order to compute the likelihood approximation *ℓ*^*N*^(*θ*), which is necessary to obtain the weights in the NPMC algorithm and the acceptance probability in the PMH method, we run a bootstrap filter with *N* = 100 particles. We recall that the latter algorithm is detailed in the supplementary material S1 Appendix.

**Fig 3:**
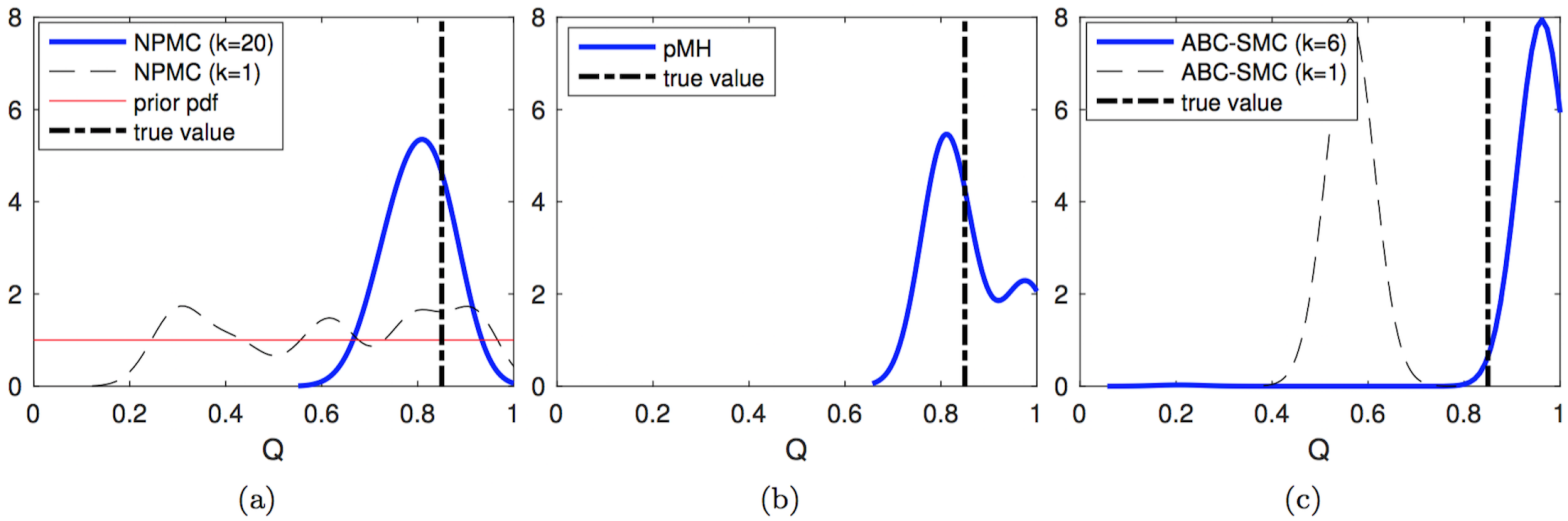
Estimated posterior pdf of *Q*. Posterior pdf’s computed from the outcome of: (a) the NPMC algorithm with *M* = 200 samples per iteration, over 20 iterations, (b) the PMH algorithm with scale parameter *σ* = 0.04 generating a chain of length *L* = 4,000 elements, and (c) the ABC-SMC method with 5 stages, tolerances *ϵ*_1:5_ = {3.0, 2.4, 2.3, 2.2, 2.1} and 800 accepted samples per stage. The true parameter value is shown with a vertical dashed line. For the NPMC method, the prior pdf, the pdf after the first iteration and the pdf after the last iteration are shown. For the ABC-SMC scheme, the pdf’s computed from the first and last population are displayed.

We first consider the estimation of the posterior probability density functions (pdf’s) of the unknown parameters given the ground truth signal and the sequence of observations produced as described above. Fig 3 shows the kernel density estimators obtained for the *Q* parameter *in a single typical run* of the PMH (left), PMH (middle) and ABC-SMC (right) algorithms. In the three plots, the actual value of the parameter *Q* is indicated with a dashed vertical line. For the NPMC algorithm with *N* = 200 samples per iteration, we additionally plot

- the a priori uniform pdf (solid red line)
- the pdf estimated after the first iteration of the algorithm (dashed black line), and
- the pdf estimated after 20 iterations of the algorithm (solid blue line).

For the PMH algorithm, the figure shows the posterior pdf estimate computed from the last 2,000 samples of a chain of total length *L* = 20 × 200 = 4,000, so that the complexity is approximately the same as with the NPMC scheme. The last plot shows the results for the ABC-SMC method with 5 populations. In particular, the dashed black line displays the pdf estimate from the first population and the solid blue line corresponds to the density estimate obtained from the fifth population. All pdf estimates have been obtained via the ksdensity function of Matlab R2016a, which uses a Gaussian kernel and calculates the bandwidth automatically as a function of the number of available samples.

We observe how the pdf estimate produced by the NPMC method improves significantly from the first to the last (20-th) iteration. Both the NPMC and PMH methods yield final density estimates which fit the ground truth value fairly accurately: both density estimators have their mode close to the true value of *Q* and the probability is also rather tightly concentrated around this value (it falls toward 0 quickly). For the ABC-SMC algorithm, there is a also an improvement from the first to the last population, but the final density estimate still leaves the true value of *Q* on the left tail of the function.

**Fig 4:**
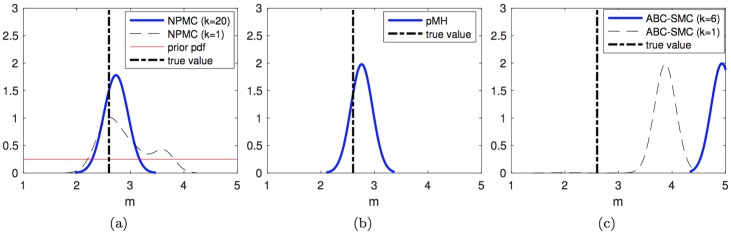
Estimated posterior pdf of m. Posterior pdf’s computed from the outcome of: (a) the NPMC algorithm with *M* = 200 samples per iteration, over 20 iterations, (b) the PMH algorithm with scale parameter *σ* = 0.04 generating a chain of length *L* = 4,000 elements, and (c) the ABC-SMC method with 5 stages, tolerances *ϵ*_1:5_ = {3.0, 2.4, 2.3, 2.2, 2.1} and 800 accepted samples per stage. The true parameter value is shown with a vertical dashed line. For the NPMC method, the prior pdf, the pdf after the first iteration and the pdf after the last iteration are shown. For the ABC-SMC scheme, the pdf’s computed from the first and last population are displayed.

**Fig 5:**
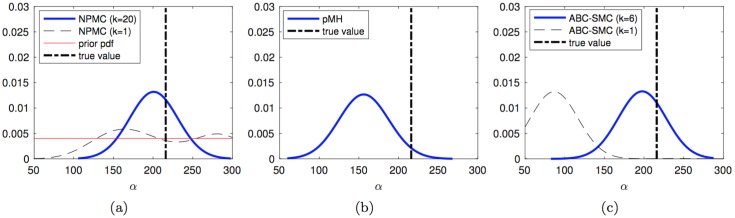
Estimated posterior pdf of *α*. Posterior pdf’s computed from the outcome of: (a) the NPMC algorithm with *M* = 200 samples per iteration, over 20 iterations, (b) the PMH algorithm with scale parameter *σ* = 0.04 generating a chain of length *L* = 4,000 elements, and (c) the ABC-SMC method with 5 stages, tolerances *ϵ*_1:5_ = {3.0, 2.4, 2.3, 2.2, 2.1} and 800 accepted samples per stage. The true parameter value is shown with a vertical dashed line. For the NPMC method, the prior pdf, the pdf after the first iteration and the pdf after the last iteration are shown. For the ABC-SMC scheme, the pdf’s computed from the first and last population are displayed.

**Fig 6:**
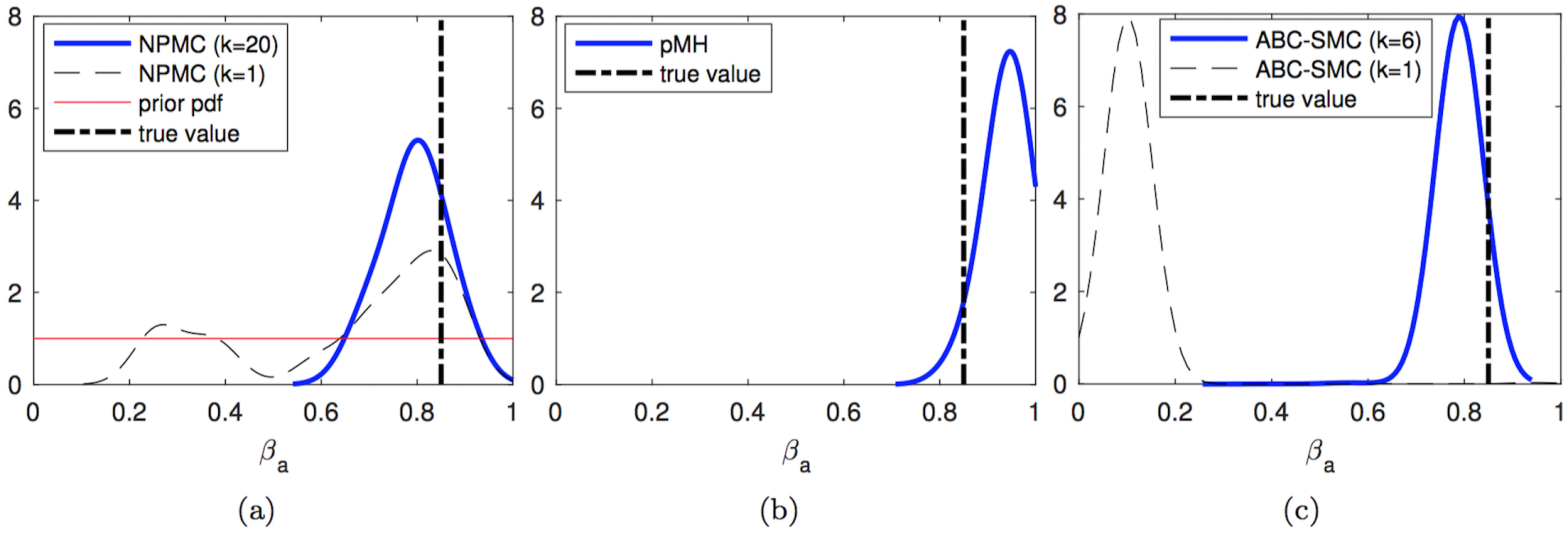
Estimated posterior pdf of *β*_*a*_. Posterior pdf’s computed from the outcome of: (a) the NPMC algorithm with *M* = 200 samples per iteration, over 20 iterations, (b) the PMH algorithm with scale parameter *σ* = 0.04 generating a chain of length *L* = 4,000 elements, and (c) the ABC-SMC method with 5 stages, tolerances *ϵ*_1:5_ = {3.0, 2.5, 2.4, 2.3, 2.2, 2.1} and 800 accepted samples per stage. The true parameter value is shown with a vertical dashed line. For the NPMC method, the prior pdf, the pdf after the first iteration and the pdf after the last iteration are shown. For the ABC-SMC scheme, the pdf’s computed from the first and last population are displayed.

Figs 4–6 display the kernel density estimators, computed in the same manner, for the unknown parameters m, *α* and *β*_*a*_. The results are similar as for parameter *Q*. Both the NPMC and PMH algorithms yield comparably fit pdf estimators with a similar computational cost of 20 × 200 = 4,000 random draws in total. The density estimators produced by the NMPC algorithm improve significantly through the iterations. The ABC-SMC algorithm yields similarly good density estimates for *α* and *β*_*a*_, with considerable improvement from the first to the fifth population as well, but there is a poor outcome for parameter m. In this latter case, the density estimate after the first iteration is actually better than the last one (after the fifth iteration). While this effect does not necessarily occur in every simulation run, we show below that the ABC-SMC method indeed has the poorest average performance of the three schemes.

From the plots in Figs 3–6, we observe that the probability mass of the pdf estimates tends to concentrate around the region where the actual parameter value is located, e.g., for the NPMC and PMH algorithms and parameter *Q*, or for the ABC-SMC algorithm and parameter *α*. However, there is no perfect match (the mode of the estimated pdf does not exactly coincide with the ground-truth value of the parameter) and we can also see some cases, e.g., the density estimate produced by the ABC-SMC scheme for parameter m, where the probability mass is placed far away from the true parameter value.

To explain these mismatches, let us recall that the approximate statistics generated by the NPMC and PMH algorithms converge to the true value of these statistics as the computational effort is increased. For example, if we are interested in the posterior mean of *θ*, then the NPMC estimate has the form

$$\begin{array}{c} {\hat{\theta }}_{k}^{M}=\sum_{i=1}^{M}w_{k}^{i}\theta_{k}^{i} \end{array}$$ (31)

after the *k*-th iteration. It can be proved [26] that $\lim_{M\to \infty }{\hat{\theta }}_{k}^{M}=E\left[\theta |y\right]$, where *E*[*θ*|**y**] is the expected value of the parameter vector *θ* conditional on the observations **y**. However, depending on the available data (especially, the dimension of vector **y**), the posterior mean *E*[*θ*|**y**] can be significantly different from the *true* value of *θ* used to generate the synthetic data.

**Fig 7:**
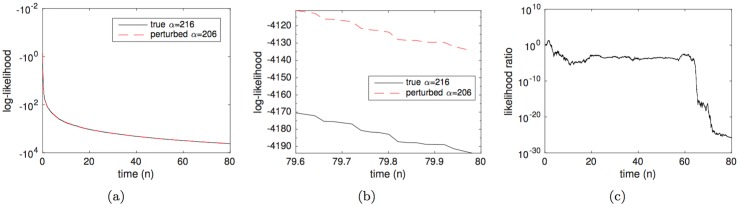
Comparison of the approximate likelihood of the true parameter vector θ_*_ = [0.85, 2.6, 216, 0.85] and perturbed version $\theta^{\prime }=\left[0.85,2.6,\underline{206},0.85\right]$. (a) Approximate log-likelihoods, *ℓ*(**y**_1:*n*_|θ_*_) and *ℓ*(**y**_1:*n*_|*θ*′) versus time *n*. (b) Zoom of plot (a) for a shorter time interval, showing that the perturbed parameter *θ*′ yields a higher likelihood for the observation sequence y generated in this computer experiment. (c) The likelihood ratio $\frac{\ell^{N}\left(y_{1:n}|\theta_{*}\right)}{\ell^{N}\left(y_{1:n}|\theta^{\prime }\right)}$ versus time *n*, showing that *ℓ*^*N*^(**y**_1:*n*_|*θ*′) > *ℓ*^*N*^(**y**_1:*n*_|θ_*_).

A simple way to illustrate this issue is to approximate the likelihood of two different parameter vectors, say $\theta_{*}=\left[Q_{*},m_{*},\alpha_{*},\beta_{a*}\right]=\left[0.85,2.6,216,0.85\right]$ the ground truth, and *θ*′ = θ_*_ + [0, 0, −10, 0] a mismatched version, and see that, for a common and fixed observation vector, they are approximately the same (actually, *ℓ*^*N*^(*θ*′) > *ℓ*^*N*^(θ_*_), even if the difference is small). This is shown in Fig 7, which, for a fixed sequence **y** = {**y**_1_, **y**_2_, …, **y**_*n*_, …}, depicts the approximate log-likelihood log(*ℓ*^*N*^(**y**_1:*n*_|θ_*_)) and log(*ℓ*^*N*^(**y**_1:*n*_|*θ*′)) versus *n*. The number of particles in the BF is set to *N* = 600 in this case to ensure that we obtain low-variance estimates.

Next, we aim at a comparison of the three parameter estimation schemes in terms of their normalised mean square error (NMSE). Assume that we run *J* independent simulations and we let

$$\begin{array}{c} \hat{\theta }\left(j\right)=\left(\hat{Q}\left(j\right),\hat{m}\left(j\right),\hat{\alpha }\left(j\right),{\hat{\beta }}_{a}\left(j\right)\right) \end{array}$$ (32)

be the estimate of the unknown parameter vector output by a given algorithm in the *j*-th simulation trial. Then, we calculate the empirical NMSE for that estimation algorithm as

$$\begin{array}{c} \text{NMSE}=\frac{1}{4J}\sum_{j=1}^{J}\left(\frac{{\left(\hat{Q}\left(j\right)-Q_{*}\right)}^{2}}{Q_{*}^{2}}+\frac{{\left(\hat{m}\left(j\right)-m_{*}\right)}^{2}}{m_{*}^{2}}+\frac{{\left(\hat{\alpha }\left(j\right)-\alpha_{*}\right)}^{2}}{\alpha_{*}^{2}}+\frac{{\left({\hat{\beta }}_{a}\left(j\right)-\beta_{a*}\right)}^{2}}{\beta_{a*}^{2}}\right), \end{array}$$ (33)

i.e., the NMSE is the average empirical quadratic error, normalised per parameter.

The three schemes for Bayesian parameter estimation admit some tuning that may affect their performance. For the NPMC algorithm, there is the choice of the proposal pdf and the clipping parameter *M*_*c*_. For all the experiments, we assume that the *k*-th iteration proposal *q*_*k*_, is Gaussian (with mean and covariance computed using the TIWs from the (*k* − 1)-th iteration) and $M_{c}=\left\lfloor \sqrt{M}\right\rfloor$, which is its maximum admissible value that guarantees asymptotic convergence [11]. The number of samples in the BF (to compute the likelihood *ℓ*^*N*^(**y**|*θ*)) is set to *N* = 100, which has been found to yield a good trade-off between computational cost and accuracy in the calculation of the weights.

Based on the results from [8] and a number of additional simulation trials (not shown in the paper) we have selected a configuration for the ABC-SMC algorithm with 5 populations, tolerances *ϵ*_1:5_ = {3.0, 2.4, 2.3, 2.2, 2.1} and a target of *J* accepted samples per population (with take *J* = 200, 800 and 1,600 for different experiments). This is the configuration, with *J* = 800 samples, already used for the pdf estimates shown in Figs 3–6. We have tried other sequences of tolerances with very similar results. The configuration of choice yields a computational cost which is similar to the NPMC algorithm with *M* = 200 and *K* = 20, and the PMH scheme with *L* = 4,000 entries in the Markov chain. The distance *d*(**y**, **y**(*θ*′)) is quadratic, namely,

$$\begin{array}{c} d\left(y,y\left(\theta^{\prime }\right)\right)=\frac{1}{K}\sum_{n=0}^{K-1}{∥y_{n}-y_{n}\left(\theta^{\prime }\right)∥}^{2} \end{array}$$ (34)

and, since the observations vectors are 2 × 1 and the observation noise has variance $\sigma_{y}^{2}=1$, the expected distance is $E\left[d\left(y,y\left(\theta^{\prime }\right)\right)\right]\geq 2\sigma_{y}^{2}=2$ even in the case of perfect parameter estimation and perfect initialisation. Therefore, the tolerance of the last population should always be greater than 2.

As for the PMH algorithm, we have assumed that the candidate samples ${\tilde{\theta }}_{m}$ are drawn from a truncated Gaussian kernel $M(\theta_{m}|\theta_{m-1})$, constructed from the Gaussian distribution with mean *θ*_*m*−1_, covariance matrix

$$\begin{array}{c} \Sigma =\sigma^{2}\left(\begin{array}{cccc} 0.01 & 0 & 0 & 0\\ 0 & 100 & 0 & 0\\ 0 & 0 & 0.01 & 0\\ 0 & 0 & 0 & 0.01 \end{array}\right), \end{array}$$ (35)

and support restricted to the set S=(0,1)×(1,5)×(50,300)×(0,1). The likelihood needed to determine the acceptance probability of a sample $\tilde{\theta }$ is approximated as $\ell^{N}\left(y|\tilde{\theta }\right)$, using the BF with *N* = 100 particles, the same as for the NPMC scheme. Note that, given **Σ** above, the parameters are sampled independently with variance proportional to their support sets (the support of *α* being much larger). The scale parameter *σ*^2^ has a direct impact on the performance of the PMH method and, hence, we have carried out computer simulations to optimise it.

**Fig 8:**
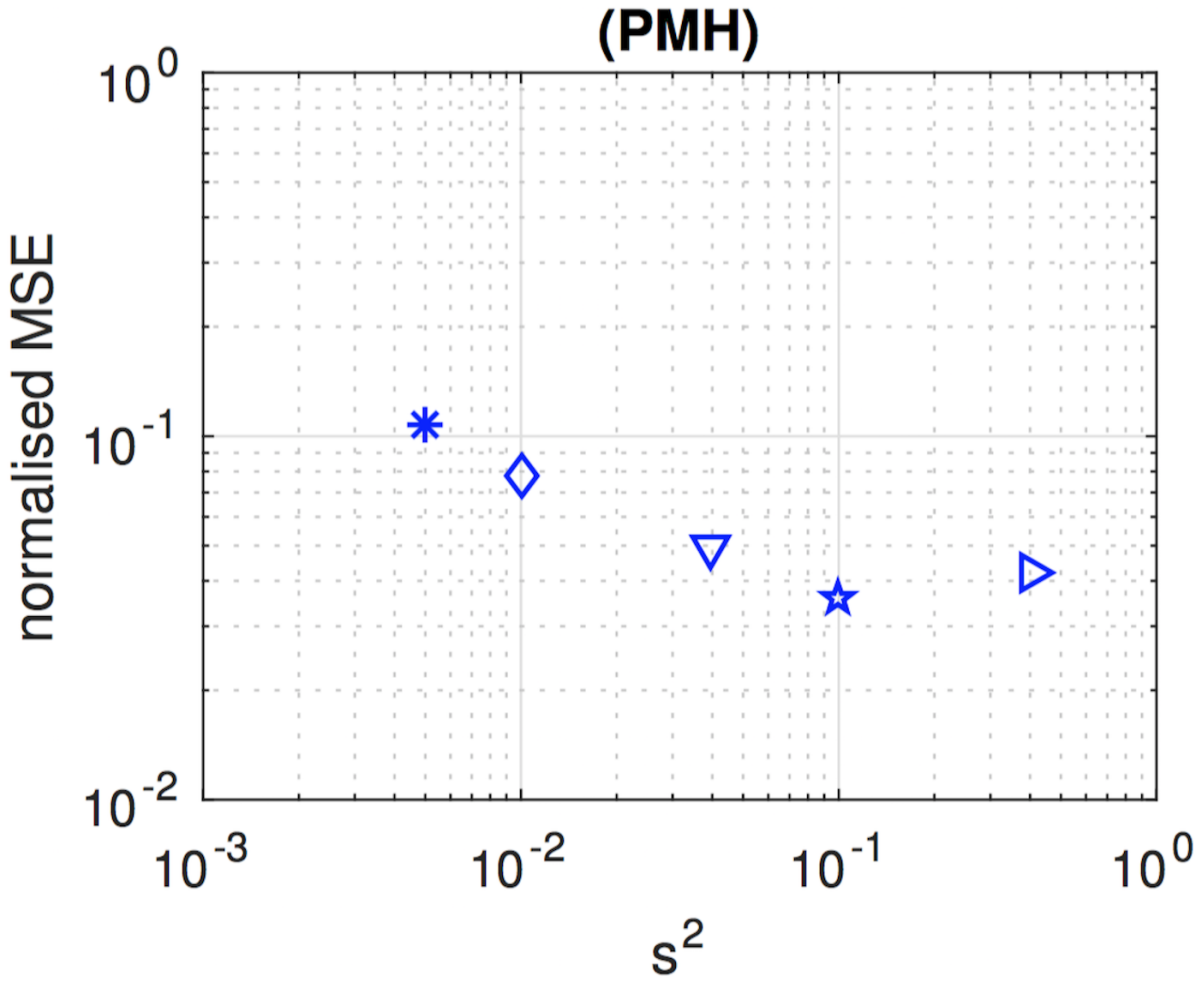
Performance of the PMH algorithm with varying scale parameter. NMSE attained by the PMH algorithm with scale parameter *s*^2^ ∈ {0.005, 0.010, 0.040, 0.100, 0.400}. This parameter controls the variance of the truncated Gaussian kernel used to generate candidate values of the Markov chain. The accuracy of the algorithm is sensitive to the choice of *s*^2^ as it determines the pace of mixing [15] of the Markov kernel $M(\theta_{m}|\theta_{m-1})$.

Fig 8 shows the variation of the NMSE, for the PMH algorithm with *L* = 4,000, as we increase the value of the scale parameter from *σ*^2^ = 0.005 up to *σ*^2^ = 0.4. The plot has been obtained as an average of 40 independent simulation runs. For each run, a different sequence of state realisations and observations is generated, and then the PMH algorithm is run five times for the same observations with different values of *σ*^2^. The figure shows that the NMSE keeps improving as we increase *σ*^2^ until it worsens for *σ*^2^ = 0.4. From this experiment, we choose *σ*^2^ = 0.1 as the scale parameter for the PMH scheme that we compare with the other two algorithms.

**Fig 9:**
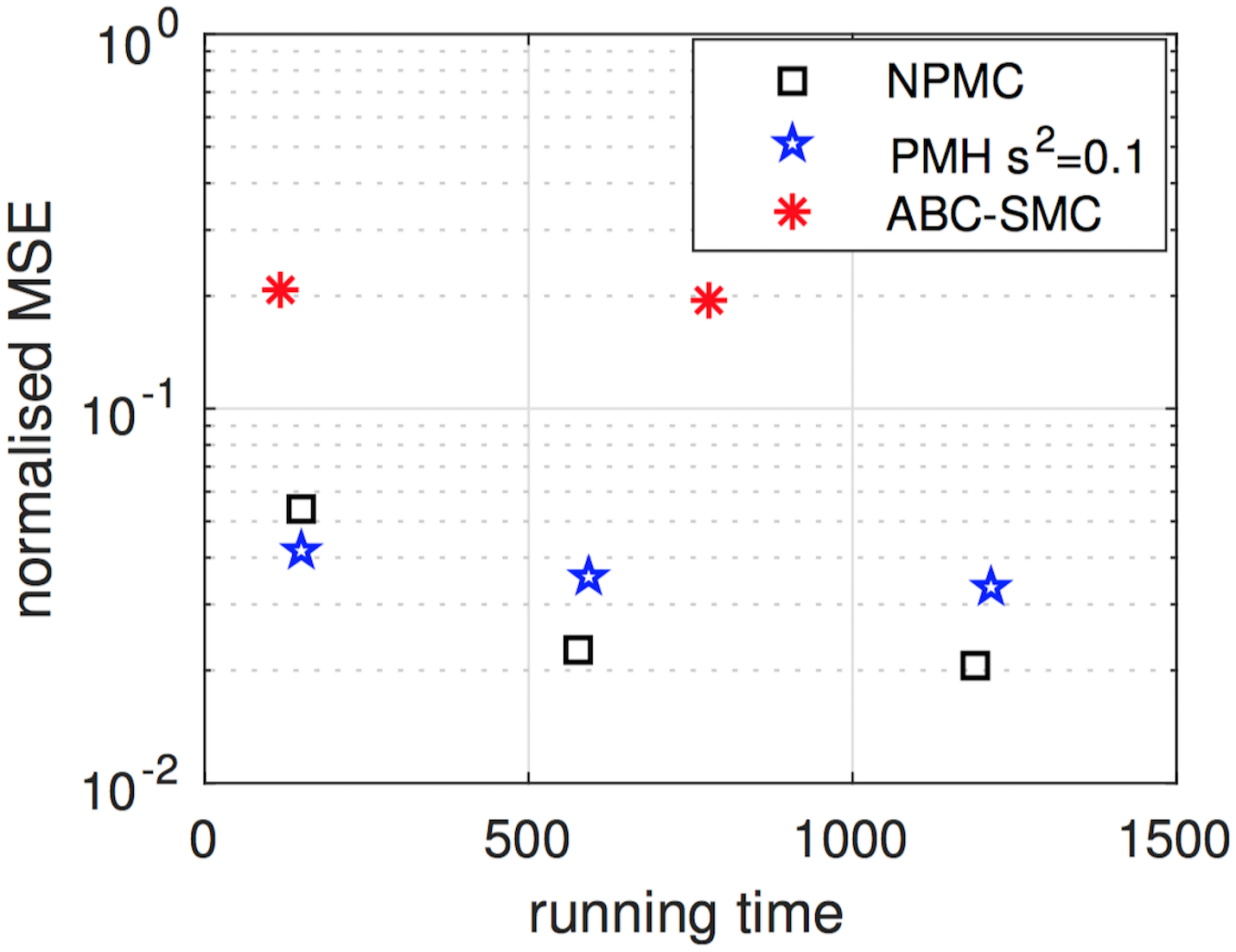
Normalised MSE of the PMH, NPMC and ABC-SMC algorithms versus running time. The figure displays the average normalised MSE per parameter attained by the NPMC scheme (with *M* = 50, 200 and 400 samples per iteration, after *K* = 20 iterations), the PMH algorithm with chains of length *L* = 1,000, *L* = 4,000 and *L* = 8,000, and scale parameter *s*^2^ = 0.1, and the ABC-SMC method with *J* = 200 and *J* = 800 samples per population. The lowest error is achieved by the NPMC algorithm, which demands a running time slightly shorter than the PMH schemes and much shorter than the (average) running time of the ABC-SMC method. Time is given in minutes.

Fig 9 displays the NMSE versus running time (in minutes) attained by

- the NPMC scheme with *K* = 20 iterations and *M* = 50, 200, 400 samples per iteration,
- the PMH method with chain lengths of *L* = 50 × 20 = 1,000, *L* = 200 × 20 = 4,000 and *L* = 400 × 20 = 8,000, and scale parameter *σ*^2^ = 0.1, and
- the ABC-SMC algorithm with tolerances *ϵ*_1:5_ = {3.0, 2.4, 2.3, 2.2, 2.1} and targets of *J* = 200 and *J* = 800 samples per population.

The values of *M*, *K*, *L* and *J* are chosen to attain comparable running times for the three methods. Note, however, that while the running times for the PMH and NPMC algorithms are deterministic given the parameters *L*, *M* and *K*, the running time of the ABC-SMC algorithm is random (even for fixed *J*), since an a priori unknown proportion of samples is expected to be rejected. Therefore, the times shown in the figure for this algorithm are an average (over 40 independent simulations). Moreover, to avoid that the ABC-SMC procedure gets stalled at any particular population, a limit of 4,000 × *J* random draws per population has been imposed (which amounts to a minimum acceptance rate of ≈25 × 10^−5^). If this limit is reached, the population is taken as complete even if containing less than *J* samples. Even with this limitation, the ABC-SMC has the largest (average) running time, and hence the highest computational cost, of all the algorithms tested.

The figure shows that the PMH algorithm attains the best performance when the computational budget is kept minimal (*M* = 50 and *L* = 1,000 for the NPMC and PMH algorithms, respectively) but the NPMC scheme yields the overall best results (for comparable running times) as the computational effort is moderately increased. Both the NPMC and PMH schemes outperform the ABC-SMC method clearly for this example. The most efficient choice for this experiment appears to be the NPMC scheme with *M* = 200 samples per iteration and *K* = 20 iterations. Note that the error decreases only slightly as we increase the number of samples to *M* = 400, even if the running time is duplicated.

**Fig 10:**
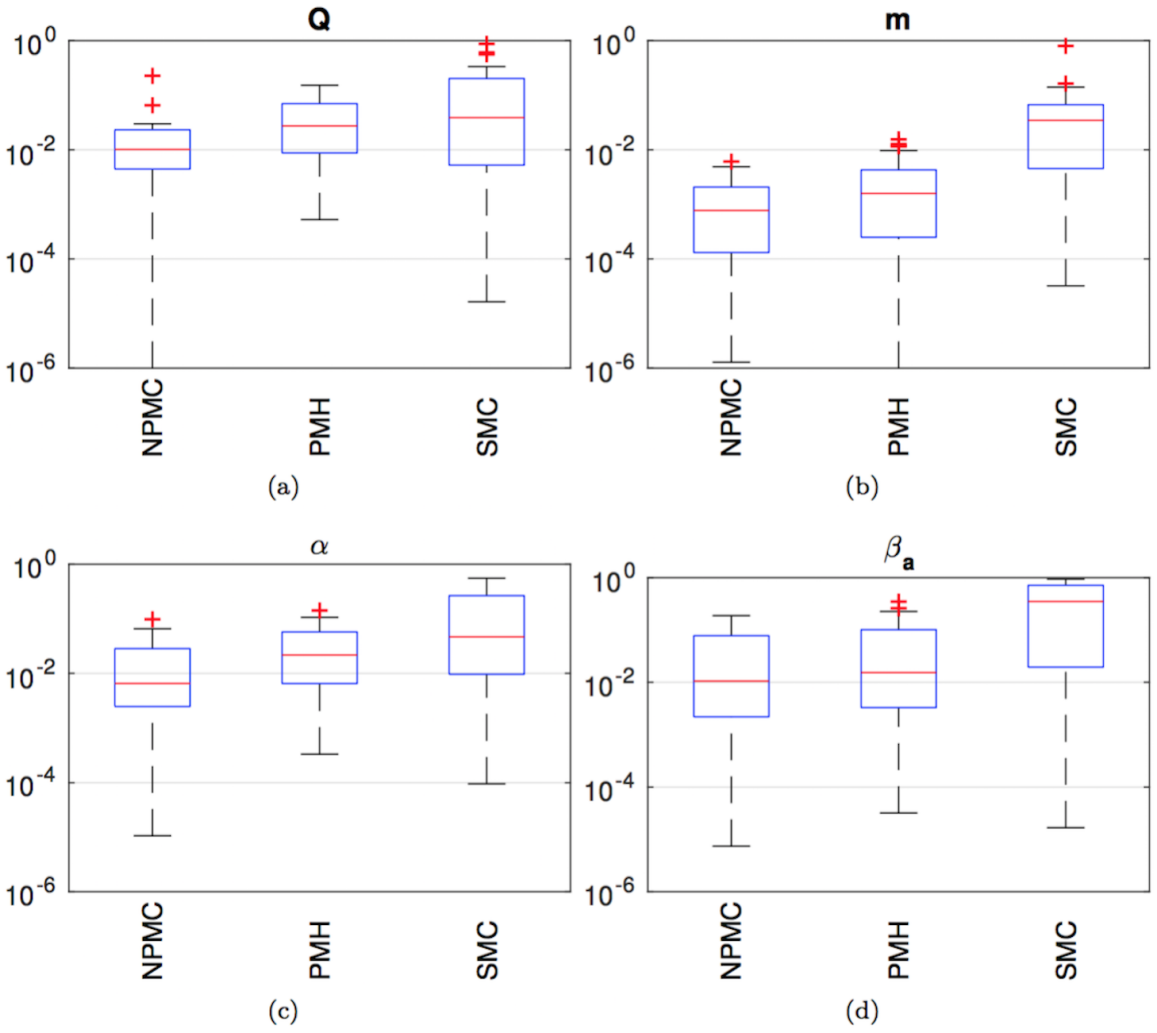
Box plots of the empirical NMSE for the NMPC algorithm with *M* = 200 and *K* = 20, the PMH scheme with scale factor *σ*^2^ = 0.1 and *L* = *M* × *K* = 4,000 samples and the ABC-SMC method. (a) Parameter *Q*. (b) Parameter m. (c) Parameter *α*. (d) Parameter *β*_*a*_. For each box, the red central mark is the median NMSE, the edges of the blue box are the 25th and 75th percentiles, the black whiskers extend to the most extreme data-points which are not considered outliers. Outliers are plotted individually as red crosses.

Fig 10 displays box-plots of the NMSE outcomes of the same 40 independent simulation runs carried out to obtain Fig 9, but only for the NPMC (*M* = 200 samples), PMH (*L* = 4,000, scale factor *σ*^2^ = 0.1) and ABC-SMC (*J* = 800) algorithms, which demand comparable running times. For each box, the red central mark is the median, the edges of the blue box are the 25th and 75th percentiles, the black whiskers extend to the most extreme data-points which are not considered outliers, and the outliers are plotted individually as red crosses. A point is taken to be an outlier if it is larger than $q_{75\%}+\frac{3}{2}\left(q_{75\%}-q_{25\%}\right)$, where $q_{75\%}$ and $q_{25\%}$ are the 75% and 25% percentiles, respectively.

We observe four plots in Fig 10, labeled (a), (b), (c) and (d), and corresponding to the parameters *Q*, m, *α* and *β*_*a*_, respectively. The outcomes are similar for all four parameters. The NMPC and PMH method perform similarly, with the median of the NPMC errors being slightly lower for all parameters and the dispersion ($|q_{75\%}-q_{25\%}|$) being similar or slightly smaller for NPMC compared to PMH. The ABC-SMC algorithm displays a consistently higher median NMSE and a slightly larger dispersion than the other two schemes.

The purpose of introducing a stochastic model of the coupled repressilator system is, as explained in the Introduction to this paper, twofold. On one hand, it provides a formalism to account for uncertainties in the design and realisation of the system. On the other hand, it sets up a probabilistic framework that enables the application of the NPMC and PMH computational schemes (and possibly other Bayesian techniques) for parameter estimation. From the latter point of view, it is of interest to test whether these methods are still useful when the underlying signals are produced by a deterministic, rather than stochastic model. With this aim, we have repeated the same computer experiments as in Figs 8–10 when the realisations of the state variables (*a*_*i*_, *b*_*i*_, *c*_*i*_, *A*_*i*_, *B*_*i*_, *C*_*i*_) are produced by the deterministic system given by Eqs (9)–(15) with null variances (i.e., *σ*_*a*_ = *σ*_*b*_ = *σ*_*c*_ = *σ*_*A*_ = *σ*_*B*_ = *σ*_*C*_ = *σ*_*S*_ = 0). We still generate noisy observations, with $\sigma_{y}^{2}=1$ as in the previous experiments, to account for observational noise.

**Fig 11:**
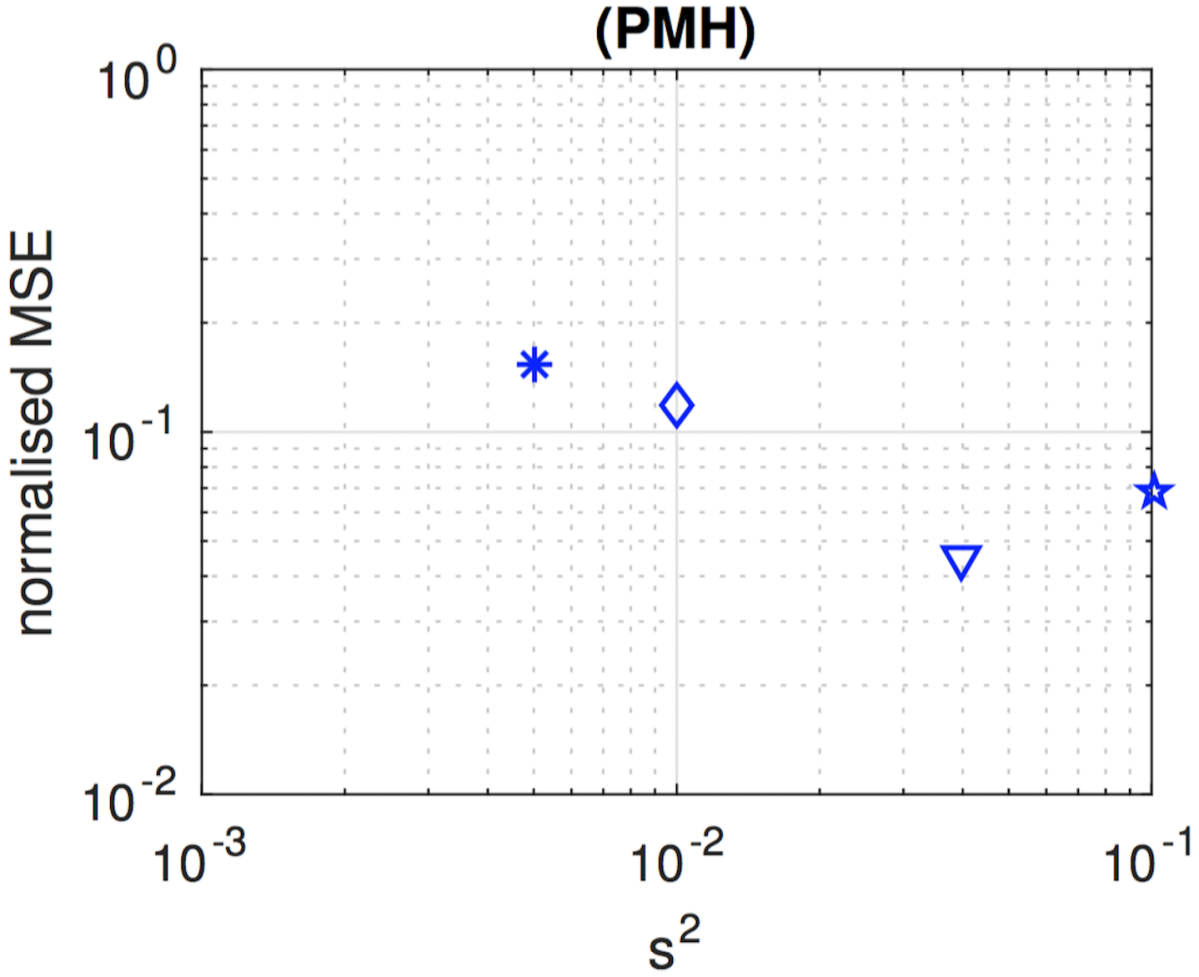
Performance of the PMH algorithm with varying scale parameter for the deterministic coupled repressilator with noisy observations. Empirical NMSE attained by the PMH algorithm with scale parameter *s*^2^ ∈ {0.005, 0.01, 0.04, 0.1}. This parameter controls the variance of the truncated Gaussian kernel used to generate candidate values of the Markov chain. Compared to the case with stochastic trajectories, the best performance is attained with a smaller value of *s*^2^ (hence, by a Markov kernel with less variance). Results obtained averaging 40 independent simulation runs.

Fig 11 shows the NMSE achieved by the PMH method with varying scale parameter *σ*^2^ for the deterministic coupled repressilator with noisy observations. Compared to the results with the stochastic system, we observe that the best performance is attained with a smaller kernel variance, namely with a scale factor *σ*^2^ = 0.04 (versus *σ*^2^ = 0.1 in Fig 8).

**Fig 12:**
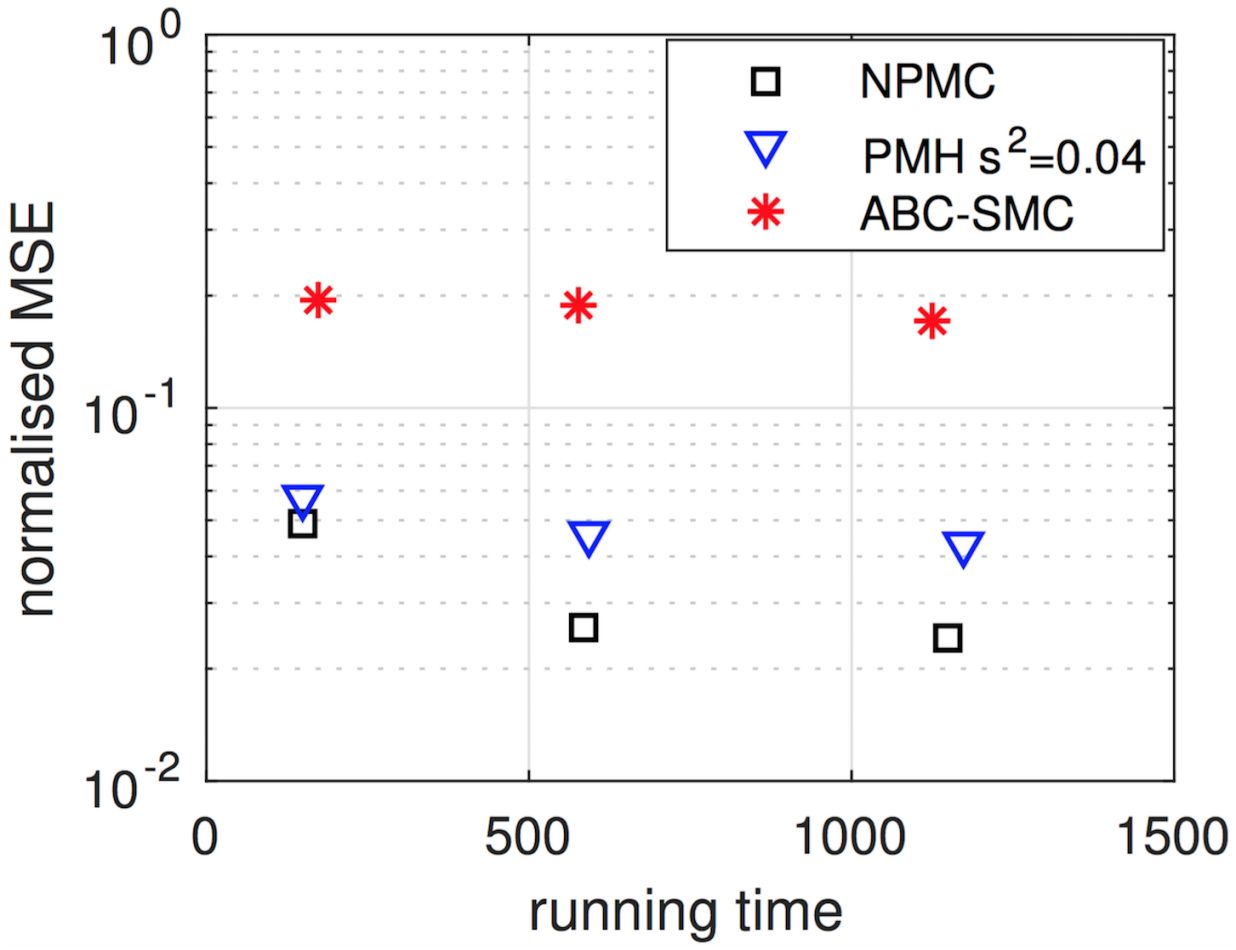
NMSE of the PMH, NPMC and ABC-SMC algorithms versus running time, for noiseless trajectories. The figure displays the average NMSE per parameter attained by the NPMC scheme (with *M* = 50, 200 and 400 samples per iteration, after *K* = 20 iterations), the PMH algorithm with scale parameter *s*^2^ = 0.04 (optimised for this scenario) and chain lengths *L* = 1,000, *L* = 4,000 and *L* = 8,000, and the ABC-SMC method with *J* = 200, 800 and 1,600. The ground truth signal is generated from a deterministic coupled repressilator system, although the observations are noisy. The lowest error is achieved by the NPMC algorithm, which demands a running time slightly shorter than the PMH schemes and slightly longer than the (average) running time of the ABC-SMC method. Time is given in minutes.

Fig 12 displays the empirical NMSE of the competing algorithms when the state trajectories are realisations of the deterministic coupled repressilator model. As before, we compare the NPMC algorithm with *M* = 50, 200 and 800 samples per iteration (and *K* = 20 iterations), the PMH algorithm with chain lengths *L* = 1,000, *L* = 4,000 and *L* = 8,000, and scale parameter *σ*^2^ = 0.04 (optimised for this scenario) and the ABC-SMC algorithm with *J* = 200, 800 and 1,600 samples per population, and otherwise the same configuration as for the simulations with the stochastic model.

Although the results are similar to Fig 9 (corresponding to the stochastic model), there are some significant differences in Fig 12. First, we observe that the average running time of the ABC-SMC method (≈580 minutes for *J* = 800) reduces considerably and becomes slightly smaller than the running time of the PMH algorithms (≈596 minutes for *L* = 4,000) and the NPMC scheme (with *M* = 200, which takes ≈586 minutes). The average error of the ABC-SMC scheme is, however, still higher than the error of the other methods. The other relevant outcome is that the NMPC algorithm is now consistently better than the PMH method, including the case with minimum running time (which corresponds to *M* = 50 samples per iteration for the NPMC algorithm and *L* = 1,000 for the PMH scheme).

**Fig 13:**
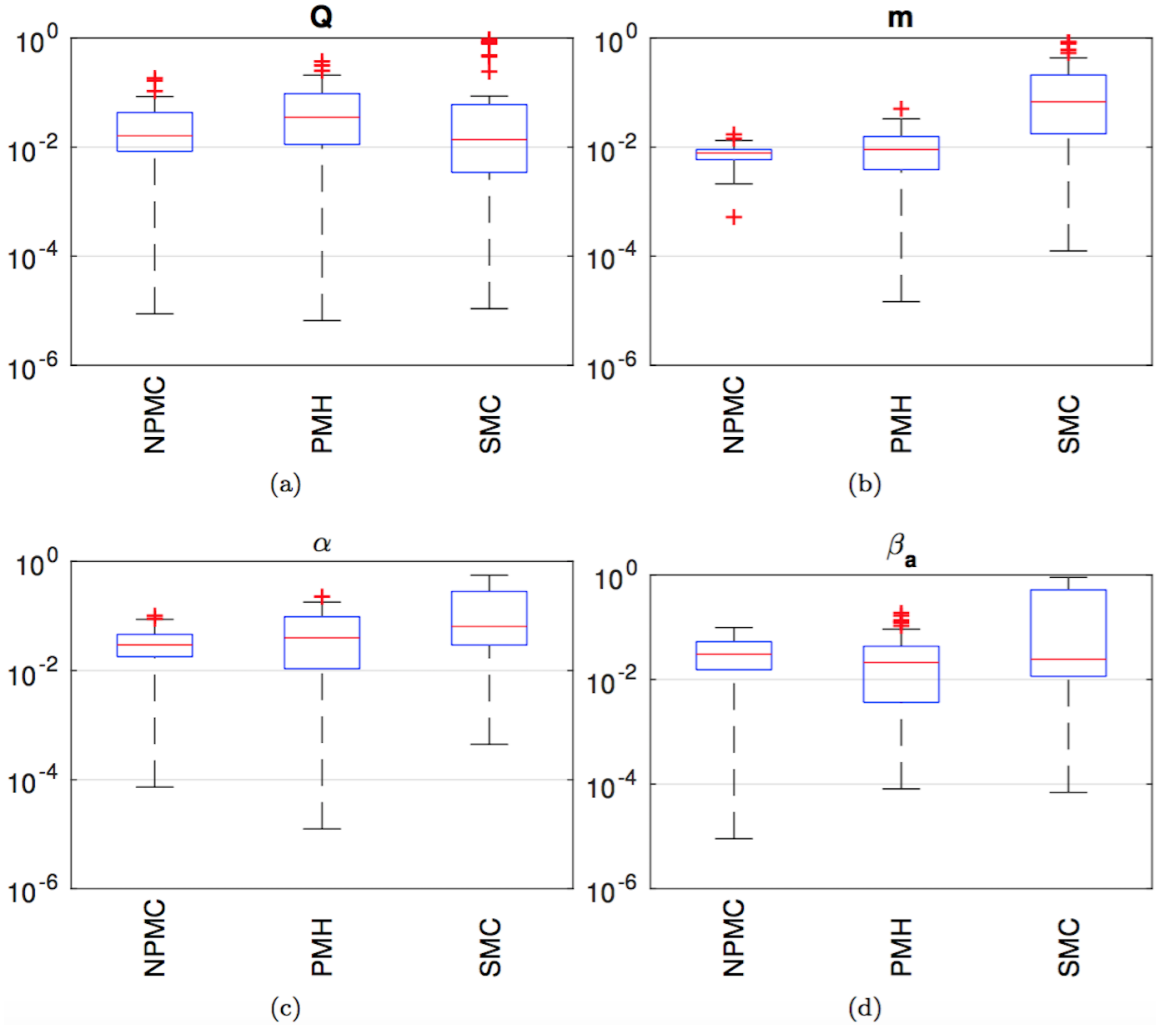
Box plots of the empirical NMSE for the NMPC algorithm with *M* = 200 and *K* = 20, the PMH scheme with scale factor *σ*^2^ = 0.1 and *L* = *M* × *K* = 4,000 samples and the ABC-SMC method. State trajectories are noiseless, generated from a deterministic coupled repressilator model. Observations are noisy. (a) Parameter *Q*. (b) Parameter m. (c) Parameter *α*. (d) Parameter *β*_*a*_. For each box, the red central mark is the median NMSE, the edges of the blue box are the 25th and 75th percentiles, the black whiskers extend to the most extreme data-points which are not considered outliers. Outliers are plotted individually as red crosses.

Finally, Fig 13 shows the box plots for the NMSE values obtained in the set of 40 independent simulation runs, for the NPMC (*M* = 200), PMH (*σ*^2^ = 0.04, *L* = 4,000) and ABC-SMC (*J* = 800) methods, which demand a similar running time. The results are plotted separately for the parameters *Q* (a), m (b), *α* (c) and *β*_*a*_ (d). We see that the median NMSE of the NPMC algorithm is slightly better than the median NMSE of the PMH scheme for all parameters except *β*_*a*_. For all four parameters, the NPMC algorithm yields a smaller dispersion of the errors.

## Conclusions

We have proposed a stochastic version of the coupled repressilator model of [5] that enables

1. a mathematically principled manner of describing experimental uncertainties in the synthesis of multicellular clocks, and
2. the design of probabilistic methods for the estimation of unknown parameters in the model, even if the underlying dynamics is chaotic, which makes the problem more challenging.

In particular, we have compared three Bayesian methods for computational model inference that enable the calculation of a posteriori probability distributions for the set of unknown parameters given a sequence of noisy observations of just two state variables. We have presented an extensive computer simulation study that illustrates the relationship between the deterministic and stochastic repressilator models and demonstrates the relative efficiency and accuracy of the nonlinear population Monte Carlo (NPMC), particle Metropolis-Hastings (PMH) and approximate Bayesian computation sequential Monte Carlo (ABC-SMC) algorithms. The best trade-off between accuracy and computational cost is attained by the NPMC algorithm, both for the deterministic and the stochastic coupled repressilator models, although the PMH scheme can attain a similar performance. The ABC-SMC method, on the other hand, has a two drawbacks. It demands knowledge of the initial condition of the system and its accuracy is directly limited by the variance of the observational noise.

## Supporting information

S1 Appendix The bootstrap filter. (PDF)

S1 Code Coding supplement (zip file). These are the matlab scripts used for the simulations in the paper. The main script is repress.m. The first part of the code includes the main simulation parameters. Notation is essentially the same as in the paper.
(ZIP)
