# Supplementary material for: A comparison of Monte Carlo-based Bayesian parameter estimation methods for stochastic models of genetic networks
Source: PLoS One. 2017 Aug 10;12(8):e0182015. doi: 10.1371/journal.pone.0182015 (PMC5552360; doi:10.1371/journal.pone.0182015)
Supplement: S1 Appendix — (PDF) [file pone.0182015.s001.pdf]

S1 Appendix. The bootstrap filter.

A Markov state-space model consists of two sequences of r.v.'s, $\{\mathbf{x}_n\}_{n\geq 0}$ and $\{\mathbf{y}_n\}_{n\geq 1}$. The first sequence, $\{\mathbf{x}_n\}$, is termed the system state. We assume it takes values on some space $\mathcal{X} \subseteq \mathbb{R}^{d_x}$, hence $\mathbf{x}_n$ is a random $d_x \times 1$ vector. The state dynamics are described by a prior probability measure $\mathcal{K}_0(\mathsf{d}\mathbf{x}_0)$ and a sequence of Markov kernels $\mathcal{K}_{n,\theta}(\mathsf{d}\mathbf{x}_n|\mathbf{x}_{n-1})$ that depend on a parameter vector $\theta \in \mathbb{R}^{d_\theta}$. In the case of the modified stochastic repressilator model, the parameter vector is $\theta = (Q, m, \beta_a, \alpha)$ and the Markov kernel $\mathcal{K}_{n,\theta}$ is given by Eq. (19). Note that we are interested on the parameters to be estimated alone. Known parameters are implicitly included in the model.

The state $\mathbf{x}_n$ cannot be observed directly. Instead, some partial noisy observations $\mathbf{y}_n$ are collected. We assume that the observations are conditionally independent given the system states and the parameter vector θ, with a conditional pdf (with respect to the Lebesgue measure) $l_{n,\theta}(\mathbf{y}_n|\mathbf{x}_n) > 0$, which depends on the parameters θ as well. For the stochastic repressilator model, the observations are given by Eq. (18), hence $l_{n,\theta}(\mathbf{y}_n|\mathbf{x}_n) = l_n(\mathbf{y}_n|\mathbf{x}_n)$ is independent of the parameter vector θ in the case of the repressilator model of interest in this paper.

The bootstrap filter (BF) [21,22] is a recursive Monte Carlo algorithm for the approximation of the sequence of posterior probability measures $\pi_{n,\theta}(\mathsf{d}\mathbf{x}_n)$, $n = 1, 2, ...$, where

- for a *given* (i.e., fixed, even if arbitrary) sequence of observations $\mathbf{y}_{1:n} = \{\mathbf{y}_1, \mathbf{y}_2, ..., \mathbf{y}_n\}$,
- and a Borel set $A \subset \mathcal{X}$,

$\pi_{n,\theta}(A)$ is the probability of the even $\mathbf{x}_n \in A$ conditional on the observations $\mathbf{y}_{1:n}$ and the parameter values given by θ. The BF with N *particles* (i.e., Monte Carlo samples) can be briefly outlined as follows.

1. ***Initialisation.*** Draw N samples $\mathbf{x}_0^1, \ldots, \mathbf{x}_0^N$ from the prior distribution $\mathcal{K}(\mathsf{d}\mathbf{x}_0)$. The particle approximation of $\pi_{0,\theta}(\mathsf{d}\mathbf{x}_0) \equiv \mathcal{K}_0(\mathsf{d}\mathbf{x}_0)$ is

$$\pi_{0,\theta}^N(\mathsf{d}\mathbf{x}_0) = \frac{1}{N}\sum_{i=1}^N \delta_{\mathbf{x}_0^i}(\mathsf{d}\mathbf{x}_0), \tag{1}$$

where $\delta_{\mathbf{x}_0^i}$ denotes the Dirac (unit) delta measure centred at $\mathbf{x}_0^i \in \mathcal{X}$.

2. ***Recursive step.*** Given the approximation $\pi_{n-1,\theta}^N(\mathsf{d}\mathbf{x}_{n-1}) = \frac{1}{N}\sum_{i=1}^N \delta_{\mathbf{x}_{n-1}^i}(\mathsf{d}\mathbf{x}_{n-1})$, take the following steps:

(a) Randomly propagate each particle using the Markov kernel in the model, i.e., draw $\tilde{\mathbf{x}}_n^i$ from $\mathcal{K}_{n,\theta}(\mathsf{d}\mathbf{x}_n|\mathbf{x}_{n-1}^i)$, $i = 1, ..., N$.

(b) Compute IWs, $\tilde{u}_n^i = l_{n,\theta}(\mathbf{y}_n|\mathbf{x}_n^i)$, for $i = 1, ..., N$, and

(c) normalise them as

$$u_n^i = \frac{\tilde{u}_n^i}{\sum_{j=1}^N \tilde{u}_n^j}, \quad i = 1, ..., N. \tag{2}$$

(d) Resample: draw N times independently from the discrete distribution

$$\tilde{\pi}_{n,\theta}^N(\mathsf{d}\mathbf{x}_n) = \sum_{i=1}^N u_n^i \delta_{\tilde{\mathbf{x}}_n^i}(\mathsf{d}\mathbf{x}_n) \tag{3}$$

and denote the resulting samples as $\{\mathbf{x}_n^i\}_{i=1}^N$. Construct the to *unweighted* approximation

$$\pi_{n,\theta}^N(\mathsf{d}\mathbf{x}_n) = \frac{1}{N}\sum_{i=1}^N \delta_{\mathbf{x}_n^i}(\mathsf{d}\mathbf{x}_n). \tag{4}$$

The resampling step (d) above can be implemented in a number of different ways (see [14] for a brief survey of methods). Here, for simplicity, we have adopted a scheme which is often referred to as multinomial resampling [25] but most asymptotic convergence results hold true for several other schemes as well [23].

The algorithm also produces an approximation of the predictive probability measure of $\mathbf{x}_n$ conditional on the observations $\mathbf{y}_{1:n-1}$. We denote the actual predictive measure as $\xi_{n,\theta}(\mathsf{d}\mathbf{x}_n)$ and its N-particle approximation as

$$\xi_{n,\theta}^N(\mathsf{d}\mathbf{x}_n) = \frac{1}{N}\sum_{i=1}^N \delta_{\tilde{\mathbf{x}}_n^i}(\mathsf{d}\mathbf{x}_n). \tag{5}$$

If we write $\mathbf{y} = \mathbf{y}_{1:t}$ for the complete sequence of observations, it turns out that the likelihood of the parameter vector θ, namely $\ell(\mathbf{y}|\theta)$ can actually be expressed in terms of integrals w.r.t. to the sequence of predictive distributions $\xi_{n,\theta}$. To be specific,

$$\ell(\mathbf{y}|\theta) = \prod_{k=1}^t \int l_k(\mathbf{y}_k|\mathbf{x}_k)\xi_{k,\theta}(\mathsf{d}\mathbf{x}_k) \tag{6}$$

and, therefore, the bootstrap filter yields the straightforward estimator

$$\ell^N(\mathbf{y}|\theta) = \prod_{k=1}^{t} \int l_k(\mathbf{y}_k|\mathbf{x}_k)\xi_{k,\theta}^N(\mathsf{d}\mathbf{x}_k) = \frac{1}{N^t}\prod_{k=1}^{t}\sum_{i=1}^{N} l_k(\mathbf{y}_k|\tilde{\mathbf{x}}_k^i), \tag{7}$$

which can be proved to be unbiased under very mild assumptions [**?**].
